# Hemopatch^®^ is effective and safe to use: real-world data from a prospective European registry study

**DOI:** 10.1007/s13304-022-01353-y

**Published:** 2022-08-20

**Authors:** Carlo Lombardo, Santiago Lopez-Ben, Ugo Boggi, Piotr Gutowski, Tomas Hrbac, Lukas Krska, Javier Marquez-Rivas, Domenico Russello, Elisa York, Mario Zacharias

**Affiliations:** 1 Division of General and Transplant Surgery, University of Pisa, Pisa, Italy; 2 Hepatobiliary and Pancreatic Surgery Unit, Hospital Universitari de Girona Doctor Josep Trueta, Girona, Spain; 3 Clinic for Vascular Surgery, Samodzielny Public Hospital, Szczecin, Poland; 4 Neurosurgical Clinic, University Hospital, Ostrava-Poruba, Czech Republic; 5 Department of Pediatric Surgery, Hospital Universitario Virgen del Rocio, Seville, Spain; 6 General Surgery Unit, Azienda Ospedaliera per l’Emergenza Cannizzaro, Catania, Italy; 7 Department of Surgery, Hospital La Paz, Madrid, Spain; 8 Clinic for Urology, Vivantes Auguste-Viktoria-Klinikum, Berlin, Germany

**Keywords:** Hemopatch^®^, Hemostasis, Minimally invasive procedures, Sealing

## Abstract

Surgical procedures are often impeded by bleeding and/or leakage of body fluids. These complications cannot always be resolved by conventional surgical techniques. Hemopatch^®^ is a hemostatic patch that also functions as a sealant. Here we document the effectiveness and safety of Hemopatch^®^ for routine procedures of multiple surgical disciplines. To this end, we performed a prospective, multicenter, single-arm, observational registry study. Patients were eligible if they had received Hemopatch^®^ during an open or minimally invasive procedure in one of these specialties: hepatobiliary, cardiovascular, urological, neurological/spinal, general, or lung surgery. Patients were excluded if they had a known hypersensitivity to bovine proteins or brilliant blue, intraoperative pulsatile or severe bleeding and/or infection at the target application site (TAS). The primary endpoint for intraoperative effectiveness was hemostasis assessed as the percentage of patients achieving hemostasis within 2 min and the percentage of patients achieving hemostasis without re-bleeding at the time of surgical closure. The registry enrolled 621 patients at 23 study sites in six European countries. Six hundred twenty patients had completed follow-up information. Hemostasis within 2 min was achieved at 463 (74.5%) of all 621 TASs. Hemostasis without re-bleeding was observed at 620 (99.8%) TASs. Adverse events were reported in 64 patients (10.3%). This Hemopatch^®^ registry shows that Hemopatch^®^ efficiently establishes hemostasis and sealing in a variety of surgical specialties, including minimally invasive procedures. Furthermore, we provide evidence for the safety of Hemopatch^®^ across all the specialties included in the registry. This study is registered at clinicaltrials.gov: NCT03392662.

**Supplementary Information:**

The online version contains supplementary material available at 10.1007/s13304-022-01353-y.

## Introduction

Bleeding and leakage of body fluids are among the most common surgical complications [1]. Major bleeding sites must be fixed by ligature or suture. On the other hand, minor bleedings, as well as small lymphatic and dural leaks, can be permanently addressed by topical hemostatic agents and surgical sealants. Therefore, these products have become an important means to supplement conventional techniques in an effort to reduce avoidable complications and improve patient outcomes [2, 3]. Since the first gelatin pads were available for surgical use in 1945 [4], hemostatic technology has advanced greatly. Modern hemostatic patches usually function as sealants as well, based on their mechanism of action. Among these is Hemopatch^®^, which consists of a thin, pliable collagen pad with high liquid absorption capacity, coated with a protein-reactive polyethylene glycol monomer (pentaerythritol polyethylene glycol ether tetra-succinimidyl glutarate, NHS-PEG). Covalent binding to proteins via the NHS-PEG coating induces the formation of a hydrogel that allows rapid adhesion to the target site, where the patch forms a liquid-tight barrier. The collagen matrix also promotes blood clotting. Crosslinking is facilitated by blood and other body fluids or can be achieved with sodium bicarbonate solution if Hemopatch^®^ is applied on a dry target application site [3, 5].

Several case series and smaller studies describe the effective and reliable use of Hemopatch^®^ in a variety of clinical applications, including solid organ, neurological, gastrointestinal, biliopancreatic, endocrine, urologic and cardiovascular procedures [6–18].

However, structured and comprehensive studies that report on the hemostatic effectiveness and safety of Hemopatch^®^ under routine conditions are rare [12, 18], even though the importance of real-world data in improving patient care is well known [19].

Therefore, the aim of this registry was to document the effectiveness and safety of Hemopatch^®^ for hemostasis and sealing applications in routine practice with hepatobiliary, cardiovascular, urological, neurological/spinal, general and lung surgery, including both open and minimally invasive procedures.

## Patients and methods

### Study design and participants

The Hemopatch^®^ Registry Study was a prospective, multicenter, single-arm, observational registry that enrolled patients who had received Hemopatch^®^ during surgery. It was conducted between November 2017 and January 2019 at 23 study sites in six European countries (Austria, Czech Republic, Germany, Italy, Poland, and Spain). Hemopatch^®^ was available in the participating hospitals and the surgeons were regular users of the product.

Patients of any age were eligible if they had received Hemopatch^®^ during an open or minimally invasive procedure in the following specialties: hepatobiliary, cardiovascular, urological, neurological/spinal, general or lung surgery. Following conventional surgical procedures, the decision to use Hemopatch^®^ was at the sole discretion of the surgeon in case of residual bleeding from multiple small sites and/or oozing of blood fluids. Therefore, for the purpose of this study, bleeding was defined as the residual hemorrhage after the use of mechanical hemostatic techniques (e.g. sutures, ligatures, clips) and/or hemostatic energy sources. This type of bleeding mostly corresponds to grade 1 (mild bleeding causing > 1.0–5.0 mL of blood loss per minute) and grade 2 (moderate bleeding causing > 5.0–10 mL of blood loss per minute) according to the validated bleeding severity scale [20]. Grade of bleeding was not systematically assessed in this study.

Patients were excluded if they had a known hypersensitivity to bovine proteins or brilliant blue, intraoperative pulsatile or severe bleeding at the potential target application site (TAS), or an active infection at the TAS.

The surgeons were asked to capture perioperative data on the effectiveness and safety of Hemopatch^®^ use in an electronic case report form (eCRF) and to follow the patients up for 4 weeks. The patients were assigned to six pre-defined cohorts based on the aforementioned type of surgical procedure.

### Effectiveness outcomes

According to the instructions for use (IFU), Hemopatch^®^ (Baxter Healthcare SA Zurich) can be used for hemostasis or to control leakage of other body fluids or air, if conventional surgical techniques are either ineffective or impractical. Hemopatch^®^ is applied dry, with the active surface to the tissue and approximated using dry gauze. It is recommended to hold the patch in place for two min after positioning it on the wound [5].

The primary endpoints for intraoperative effectiveness were the percentage of patients achieving hemostasis within two min and the percentage of patients achieving hemostasis without re-bleeding at the time of surgical closure. Secondary outcomes were need for blood transfusion up to 72 h, need for surgical revision, median stay in intensive care unit, and median hospital stay.

The time to hemostasis was recorded in seconds, when the surgeon judged hemostasis was achieved. In terms of leakage control, primary endpoints for intraoperative effectiveness were absence of air leakage (lung cohort) and achievement of a watertight closure, i.e. no cerebrospinal fluid (CSF) leakage after inspection according to the local standard of care (e.g. Valsalva maneuver) if the product was applied to the dura mater (neurological/spinal cohort).

Numerous variables were documented in the eCRF (supplemental file 1) to assess the intraoperative effectiveness endpoints, including the following: indication for surgery and description of procedure, target application site and tissue type, time to hemostasis (TTH), absence of air leakage, whether a watertight closure was achieved if applied to the dura mater, and if slipping of the Hemopatch^®^ occurred.

The primary postoperative effectiveness endpoints during the 4-week follow-up period were cohort-specific and are summarized in Table 1.

**Table 1:** Summary of effectiveness endpoints

Effectiveness endpoints		
Primary intraoperative effectiveness endpoints	Primary postoperative effectiveness endpoints	
Hemostasis Percentage of patients achieving hemostasis within 2 min Percentage of patients achieving hemostasis without re-bleeding at the time of surgical closure	Hepatobiliary surgery	Incidence of postoperative pancreatic fistulas, duration of bile leakage
Leakage control Lung: absence of air leak Dura: a water-tight closure was achieved, i.e. no cerebrospinal fluid (CSF) leak after inspection according to local standard of care	Lung surgery	Incidence of air leakage measured by the number of patients with chest tube drainage ≥ 5 days, and number of patients needing reinsertion of chest tube for pneumothorax
	Neurological/spinal surgery	Incidence of postoperative CSF leakage (external or internal accumulation including pseudomeningocele)
	Urological surgery	Incidence of postoperative urinary fistula formation
	General surgery	Incidence of gastrointestinal anastomosis leakage/fistula

### Safety outcomes

The safety of Hemopatch^®^ was assessed by the incidence of adverse events (AEs) related to the use of Hemopatch^®^. The primary safety endpoints were adverse events of special interest (AESI), which were defined as follows: allergic reaction to Hemopatch^®^, re-bleeding at the TAS, hematoma at the TAS, local infections at the TAS.

Secondary safety endpoints included the number of Hemopatch^®^ units applied, the use of bicarbonate in combination with Hemopatch^®^, the need for intraoperative surgical revisions due to bleeding, air or other body fluid leakage, surgery duration, postoperative transfusions up to 72 h after surgery (number, type and amount of blood product), days on intensive care unit, and length of hospital stay.

### Study size

The original study size was calculated based on published data to predict the percentage of patients per cohort that could be expected to achieve hemostasis in two minutes. A precision of 3.5% was assumed to provide a sufficiently narrow confidence interval (CI) for clinical judgment. In total, 1,194 patients (Hemopatch^®^ cases) were calculated to be required. An interim analysis was conducted, after 622 TASs in 621 patients had been included, and based on the obviously good efficacy and safety outcomes, it was decided to stop enrollment. Data were evaluated using only descriptive statistics.

### Statistical methods and data sets

Statistical analyses were mainly descriptive: continuous variables were summarized by sample size (n), mean, standard deviation (SD), median, minimum, and maximum. Frequency counts, percentages and exact 95% binomial CIs (Clopper–Pearson) for proportions were provided for categorical variables. All patients who received Hemopatch^®^ and were enrolled in the study were included in the safety analysis set (SAS). All patients with a postoperative hemostasis assessment were included in the full analysis set (FAS). Variables were summarized for all eligible patients with available data. For all key variables, the proportion of missing data was described to understand the extent to which there could have been under-reporting or bias in endpoint measurement.

Analyses were performed using SAS/GRAPH^®^ 9.4 software, SAS/STAT^®^ 14.1 software and Base SAS^®^ 9.4, SAS Institute Inc., Cary, NC, USA.

The figure was created using Adobe InDesign.

### Post hoc analyses

Post hoc analyses were performed on the total population (except for the neurological/spinal cohort) to describe the effectiveness of Hemopatch^®^ for hemostasis and to compare the effectiveness and safety of Hemopatch^®^ in patients undergoing open surgery versus minimally invasive procedures. The percentage of patients with successful hemostasis within three min was also assessed.

## Results

### Patients

**Fig. 1:**
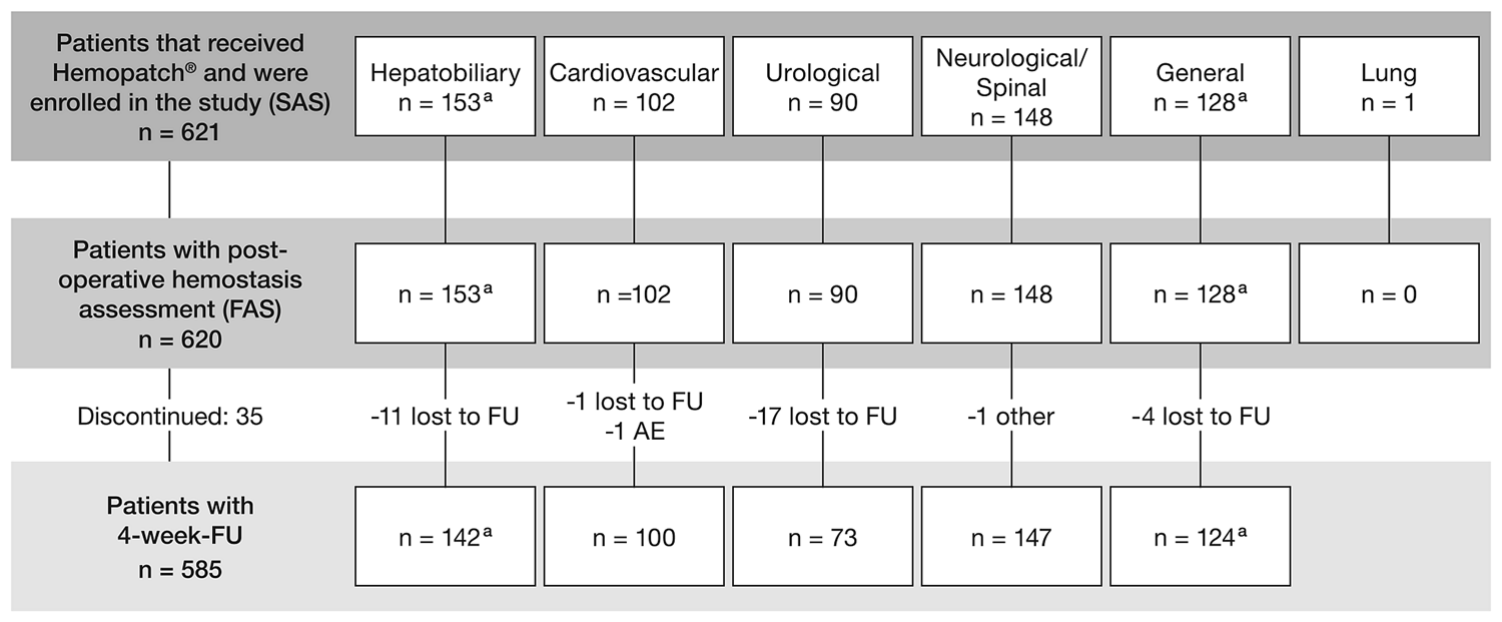
Patient disposition. *One patient was included in both cohorts since he had two target application sites: one at the mesocolon (general cohort) and one at the liver (hepatobiliary cohort). *AE* adverse event; *FAS* full analysis set; *FU* follow-up; *SAS* safety analysis set

In total, 621 patients who received Hemopatch^®^ during their surgery were enrolled in the registry (safety analysis set, SAS) at 23 study centers between 11/2017 and 01/2019. One patient was enrolled in the lung cohort but was lost to follow-up and did not receive postoperative hemostasis assessment. 620 patients who had a postoperative hemostasis assessment were included in the full analysis set (FAS). One patient received Hemopatch^®^ at two different target application sites (TASs). One patch was applied to the mesocolon, the other to the liver; this patient was included in the hepatobiliary and the general cohort. Consequently, the FAS includes 620 patients with 621 TASs. Figure 1 details patient allocations to the different cohorts according to surgical discipline.

### Patient characteristics

Table 2 summarizes the patient characteristics of the total study population and the cohorts. Except in the neurological/spinal cohort, which also included children, surgeons used Hemopatch^®^ in adult patients and the mean age was 59.4 years (SD = 17.7). A total of 73.5% of the patients underwent an open procedure, but the percentage varied considerably among the cohorts from 52.2% in the urological up to 93.9% in the neurological/spinal cohort.

**Table 2:** Patient characteristics

Number of patients	Hepato-biliary(*n* = 153^b^)	Cardio-vascular (*n* = 102)	Urological (*n* = 90)	Neuro-logical/spinal (*n* = 148)	General (*n* = 128^b^)	Lung (*n* = 1)	Total (*n* = 621)
Age, years							
Mean (SD)	65.0 (11.1)	69.4 (8.8)	65.7 (10.5)	44.1 (22.4)	58.2 (15.7)	62.0 ()	59.4 (17.7)
Median (range)	68.0 (33–91)	69.5 (47–88)	66.5 (40–82)	48.5 (1–84)	58.0 (24–95)	62.0	63 (1–95)
Gender							
Male, *n* (%)	80 (52.3)	77 (75.5)	76 (84.4)	63 (42.6)	45 (35.2)	1 (100.0)	341 (54.9)
Female, *n* (%)	73 (47.7)	25 (24.5)	14 (15.6)	85 (57.4)	83 (64.8)	0 (0.0)	280 (45.1)
Surgical approach							
Open, *n* (%)	90 (58.8)	74 (72.5)	47 (52.2)	139 (93.9)	107 (83.6)	0 (0.0)	456 (73.4)
Minimally invasive, *n* (%)	63 (41.2)	28 (27.5)	43 (47.8)	9 (6.1)	21 (16.4)	1 (100.0)	165 (26.6)
Patient with comorbidities, *n* (%)	18 (11.8)	10 (9.8)	24 (26.7)	3 (2.0)	6 (4.7)	0 (0.0)	61 (9.8)
CCI^a^, mean (SD)	4.39 (2.64)	4.20 (1.40)	4.58 (1.72)	4.00 (1.73)	3.33 (1.97)	–(–)	4.31 (2.00)

*CCI* Charlson Comorbidity Index

^a^Only patients with comorbidities

^b^One patient with two TASs was included in both the hepatobiliary and the general cohort but only counted as one patient in the total number

### Main effectiveness results—total population

Across all enrolled patients, hemostasis was achieved within two min after application of Hemopatch^®^ at 74.5% of all TASs (463 out of 621). This percentage varied from 52.0% in the cardiovascular to 91.4% in the general cohort. Hemostasis was achieved in all patients except one (99.8%) without re-bleeding at the time of surgical closure (Table 3).

**Table 3:** Effectiveness of Hemopatch

Number of patients	Hepato-biliary (*n* = 153^b^)	Cardio-vascular (*n* = 102)	Urological (*n* = 90)	Neuro-logical/-spinal (*n* = 148)	General (*n* = 128^b^)	Lung (*n* = 1)	Total (*n* = 621)
Primary intraoperative endpoints							
Hemostasis within 2 min, *n* (%)	119 (77.8)	53 (52.0)	64 (71.1)	109 (73.6)	117 (91.4)	1 (100.0)	463 (74.3)
Hemostasis without rebleeding, *n* (%)	152 (99.3)	102 (100.0)	90 (100.0)	148 (100.0)	128 (100.0)	1 (100.0)	620 (99.8)
Time to hemostasis, min							
Mean (SD)	1.80 (0.92)	2.10 (0.77)	1.78 (1.67)	1.44 (1.26)	1.25 (0.79)	0.33 ()	1.64 (1.14)
Median (range)	2.00 (0.02–5.33)	2.00 (0.20–5.00)	0.92 (0.17–8.00)	2.00 (0.00–3.00)	1.00 (0.00–5.00)	0.33 (0.33–0.33)	2.00 (0.00–8.00)
Slipping of the patch, *n* (%)	6 (3.9)	3 (2.9)	3 (3.3)	1 (0.7)	4 (3.1)	0 (0.0)	17 (2.7%)
Postoperative effectiveness endpoints^a^							
Hepatobiliary	Pancreatic fistula				*n* (%)		8 (5.2)
	Bile leakage				Median duration, days (range)		10.0 (5.00 – 48.00)
Urological	Urinary fistula				*n* (%)		2 (2.2)
Neurological/spinal	CSF leakage				*n* (%)		11 (7.4)
General	Gastrointestinal anastomosis leakage/fistula				*n* (%)		3 (2.3)
Lung	Chest tube drainage re-insertion due to pneumothorax				No follow-up		

*N/A* not applicable

^a^Percentage of occurrence was based on the total cohort size; a drill-down to procedures, where this complication could have been expected, could not be done in all cases due to limitations in documentation

^b^One patient with two TASs was included as one case in both the hepatobiliary and the general cohort. Therefore, the sum of TASs (patients) across cohorts is 622, but the total number of patients is 621

The mean TTH was 1.64 (SD = 1.14). The shortest mean TTH was reported in the general and neurological/spinal cohorts (1.25 and 1.44 min, respectively), and the longest in the cardiovascular cohort (2.1 min) (Table 3). A relevant proportion of cases therefore received effective hemostasis with Hemopatch^®^ within less than two min after application (the IFU recommends holding the product in place for two minutes after application).

Intraoperative watertight closure in the neurological/spinal cohort was documented in 131/147 patients (89.1%), where the TAS was the dura. In 15/147 (10.2%), it was documented as not applicable (N/A) and in one case (0.7%) as unknown.

Across all cohorts, slipping of the Hemopatch^®^ occurred at 17 (2.7%) TASs. The frequency of slipping varied from 0.0% in the lung cohort to 3.9% in the hepatobiliary cohort (Table 3).

The incidences of predefined, cohort-specific postoperative complications such as fistula formation or leakage events up to 4 weeks after surgery are summarized in Table 3 (postoperative effectiveness endpoints).

Only nine patients underwent a cardiovascular procedure and had chest tube drainage for 2–8 days. None of these patients required reinsertion of a chest tube, as no pneumothorax appeared postoperatively.

### Post hoc analyses

In many cases, surgeons reported a TTH of 0 min if bleeding was minor and a Hemopatch^®^ was used primarily for sealing. This was mainly the case in the neurological/spinal cohort, where a Hemopatch^®^ was applied to the dura mater in a substantial number of patients (147/148 = 99.3%).

To describe the effectiveness of the Hemopatch^®^ more reliably with an indication of hemostasis, we performed a post hoc analysis that excluded the neurological/spinal cohort. The lung cohort was also excluded since it only had one patient. Among patients of the hepatobiliary, urological, cardiovascular and general cohorts, 74.8% (353 out of 472) TASs achieved hemostasis within two min and 95.1% within three min.

### Hemopatch^®^ is effective in open surgery and minimally invasive surgery (MIS)

Among patients in the hepatobiliary, urological, cardiovascular, and general cohorts (*n* = 472), 67.2% (*n* = 317) underwent an open procedure and 32.8% (*n* = 155) underwent MIS. Hemostasis within two min was achieved by 69.72% of the patients who had open surgery, and in 85.16% of the patients who had MIS.

Hemostasis within three min was achieved in 93.38% of patients with open surgery and in 98.06% with MIS.

### Intra- and postoperative characteristics of procedures

In the majority of all procedures, surgeons used one (80.5%) or two (16.2%) patches per patient (Table 4). Replacement of a slipped patch was only necessary in three cases (two in the urological cohort and one in the hepatobiliary cohort).

**Table 4:** Intra- and postoperative characteristics of patients

Number of patients	Hepato-biliary (*n* = 153^b^)	Cardio-vascular (*n* = 102)	Urological (*n* = 90)	Neuro-logical/-spinal (*n* = 148)	General (*n* = 128^b^)	Lung (*n* = 1)	Total (*n* = 621)
Intraoperative							
Number of patches, *n* (%)							
1 patch	115^b^ (75.2)	97 (95.1)	44 (48.9)	128 (86.5)	116^b^ (90.6)	1 (100.0)	500 (80.5)
2 patches	29 (19.0)	4 (3.9)	42 (46.7)	13 (8.8)	12 (9.4)	0 (0.0)	100 (16.1)
3 patches	5 (3.3)	1 (1.0)	3 (3.3)	2 (1.4)	0 (0.0)	0 (0.0)	11 (1.8)
4 patches	4 (2.6)	0 (0.0)	1 (1.1)	3 (2.0)	0 (0.0)	0 (0.0)	8 (1.3)
5 patches	0 (0.0)	0 (0.0)	0 (0.0)	1 (0.7)	0 (0.0)	0 (0.0)	1 (0.2)
6 patches	0 (0.0)	0 (0.0)	0 (0.0)	1 (0.7)	0 (0.0)	0 (0.0)	1 (0.2)
Use of bicarbonate with Hemopatch, *n* (%)	24 (15.7)	0 (0.0)	0 (0.0)	41 (27.7)	1 (0.8)	0 (0.0)	66 (10.6)
Other hemostatic agents used, *n* (%)	3 (2.0)	0 (0.0)	0 (0.0)	14 (9.5)	4 (3.1)	(0.0)	21 (3.4)
Blood transfusions, *n* (%)	10 (6.5)	2 (2.0)	5 (5.6)	4 (2.7)	4 (3.1)	0 (0.0)	25 (4.0)
Surgical revision, *n* (%)	3 (2.0)	0 (0.0)	0 (0.0)	0 (0.0)	0 (0.0)	0 (0.0)	3 (0.5)
Postoperative							
Blood transfusion up to 72 h	10 (6.5)	5 (4.9)	3 (3.3)	1 (0.7)	8 (6.3)	0 (0.0)	27 (4.4)
Surgical revision *n* (%)	10 (6.5)	3 (2.9)	5 (5.6)	4 (2.7)	10 (7.8)	0 (0.0)	32 (5.2)
Median stay in intensive care unit, days (range)^a^	2.8 (1.1–35.6)	1.9 (1.46–3.9)	2.0 (1.72–56.3)	3.8 (1.21–62.7)	4.3 (1.67–24.8)	n.a	2.9 (1.1–62.7)
Median hospital stay, days (range)^a^	9.0 (2–54)	7.0 (2–42)	9.0 (2–56)	12.0 (2–160)	7.0 (1–69)	n.a	9.0 (1–160)

^a^Data for the patient in the lung cohort were not included as the patient was lost to follow-up

^b^One patient with 2 TASs was included as one case in both the hepatobiliary and the general cohort, but only counted as one patient in the total number

Surgeons used bicarbonate to apply Hemopatch^®^ in 66 cases. The reason for bicarbonate use was documented as ‘always use it’ in the majority of cases (62/66, 93.9%), and it was most often used in neurological/spinal interventions (41/148; 27.7%), while Hemopatch^®^ was predominantly used on the dura mater, indicating a relatively dry TAS.

A total of 4.0% of all patients received intraoperative blood transfusions. Postoperative blood transfusions were given to 4.4% of all patients. Most transfusions were administered in the hepatobiliary cohort (10 cases each intra- and postoperatively) (Table 4).

Intraoperative surgical revision was only required rarely and only in the hepatobiliary cohort. Thirty-six patients required either surgical or interventional procedures to address postoperative complications (Table 4). Table 5 summarizes the intra- and postoperative events.

**Table 5:** Safety analysis

Number of patients	Hepato-biliary (*n* = 153^b^)	Cardio-vascular (*n* = 102)	Urological (*n* = 90)	Neuro-logical/-spinal (*n* = 148)	General (*n* = 128^b^)	Lung (*n* = 1)	Total (*n* = 621)
Adverse events (AE)							No of patients (%)
Any AE, *n* (%)	25 (16.3)	15 (14.7)	7 (7.8)	5 (3.4)	12 (9.4)	0 (0.0)	64 (10.3)
Serious, *n* (%)	18 (11.8)	14 (13.7)	3 (3.3)	5 (3.4)	6 (4.7)	0 (0.0)	46 (7.4)
Deaths, *n* (%)	0 (0.0)	1 (7.1)	0 (0.0)	1 (20.0)	0 (0.0)	0 (0.0)	2 (4.3)
Hemopatch-related AE^a^	2 (1.3)	3 (2.9)	0 (0.0)	0 (0.0)	1 (0.8)	0 (0.0)	6 (1.0)
AE of special interest (AESI)							
Any AESI	4 (2.6)	3 (2.9)	0 (0.0)	2 (1.4)	4 (3.1)	0 (0.0)	13 (2.1)
Allergic reaction to Hemopatch, *n* (%)	0 (0.0)	0 (0.0)	0 (0.0)	0 (0.0)	0 (0.0)	0 (0.0)	0 (0.0)
Re-bleeding at TAS, *n* (%)	0 (0.0)	2 (2.0)	0 (0.0)	0 (0.0)	1 (0.8)	0 (0.0)	3 (0.5)
Hematoma at TAS, *n* (%)	0 (0.0)	0 (0.0)	0 (0.0)	0 (0.0)	2 (1.6)	0 (0.0)	2 (0.3)
Local infection at TAS, *n* (%)	3 (2.0)	1 (1.0)	0 (0.0)	2 (1.4)	2 (1.6)	0 (0.0)	8 (1.3)
Hemopatch-related AESI^a^	2 (1.3)	3 (2.9)	0 (0.0)	0 (0.0)	1 (0.8)	0 (0.0)	6 (1.0)

*TAS* target application site

^a^Study product-related AEs are AEs rated by the investigator as ‘probably related’ and ‘possibly related’

^b^One patient with two TASs was included as one case in both the hepatobiliary and the general cohort, but only counted as one patient in the total number

### Assessments of product and satisfaction by surgeons

Thirty-one (100%) of surgeons completed the intraoperative user survey. The hemostatic efficacy of Hemopatch^®^ was rated as “excellent” or “good” by 93.6% of the surgeons. Compared to other hemostatic patches, 51.7% of the surgeons rated the hemostatic efficacy of Hemopatch^®^ as “much better” or “better”; 41.2% rated it as “equivalent”. Overall, surgeon´s satisfaction was rated as “excellent” or “good” by all (100%) surgeons and “much better” or “better” compared to other hemostatic patches by about two-thirds (64. 6%). Nearly all surgeons (96.8%) said that they would use Hemopatch^®^ in the future.

### Safety analysis

During the 4-week follow-up period, 64 patients (10.3%) of the SAS experienced an AE, only 13 of which were classified as an AE of special interest (AESI) (Table 5). Allergic reactions to Hemopatch^®^ were not observed.

Six patients experienced adverse events that were thought to be related to Hemopatch^®^, as rated by the investigators.

The investigators reported two deaths but did not consider them to be related to Hemopatch^®^. One patient in the cardiovascular cohort died of sepsis. In the other (neurological/spinal) cohort, a patient was hospitalized with an intracerebellar hematoma and died 1 week after surgery due to worsening brain edema.

## Discussion

The Hemopatch^®^ registry is the largest study of real-world use of the Hemopatch^®^ to be carried out so far, and its effectiveness and safety have been documented in various surgical specialties. We showed that the Hemopatch^®^ is effective in achieving hemostasis and sealing in different types of tissue. The sealing capacity of Hemopatch^®^ against body fluids other than blood was clearly demonstrated. We also provided evidence for the safety and efficiency of Hemopatch^®^ in MIS.

The mean TTH was 1.64 min across all cohorts. This is in line with the IFU, which prescribes holding the patch in place for two min [21]. The TTH was slightly higher in the cardiovascular cohort (2.10 min). This result was expected due to the anticoagulation required during these procedures.

Our effectiveness results are supported by other studies addressing the effectiveness of Hemopatch^®^ in smaller groups of patients. Ulrich et al. documented successful hemostasis within two min after application of Hemopatch^®^ in 93.3% of patients undergoing different types of surgery (general, cardiac, lung, urologic, other). Furthermore, they showed that anticoagulants can reduce the hemostatic effect of Hemopatch^®^, although to a minor extent [16]. Fingerhut et al. presented a series of case reports where Hemopatch^®^ was successfully used to seal nearly all bleeding surfaces in various types of surgery [6]. Furthermore, Weltert et al. reported that Hemopatch^®^ induced hemostasis within three min in 97.6% of patients undergoing surgery for ascending aortic aneurysms, compared to only 65.8% of patients in the control group (compression with dry or wet gauze or similar) [12].

Several studies address the efficacy of another collagen-based hemostatic patch coated with human fibrinogen and human thrombin (TachoSil^®^) [22–24]. The authors reported that TachoSil^®^ hemostasis can be achieved within three min in 75% of patients undergoing cardiovascular surgery [23], and 81% undergoing hepatic resection [22], respectively. Another study documented hemostasis in kidney tumor resection to be 92% within 10 min using TachoSil^®^ [24].

In addition to its hemostatic properties, Hemopatch^®^ showed remarkable sealing capacity, thus confirming earlier findings from two case series that showed CSF leak rates of 4.5 and 5.9%, respectively when using Hemopatch^®^ as a dural sealant [10, 14]. In this study, the product was applied to the dura mater and the overall CSF leakage rate in the 147 patients was 7.5%. However, it was not clear in all cases whether the product was applied to achieve watertight dural closure or hemostasis.

In two case series involving patients after distal pancreatectomy and pancreato-duodenectomy, a promising reduction of clinically relevant postoperative pancreatic fistula (POPF) was shown [15, 25]. The first randomized controlled trial showed a significant reduction in clinically relevant POPF in the respective subgroups with hand-sewn stump closure or main duct ligation, when Hemopatch^®^ was used to seal the stump after distal pancreatectomy [18]. In our study, a precise calculation of POPF in the hepatobiliary cohort could not be made due to the limitations mentioned below.

For most procedures, one Hemopatch^®^ was adequate to achieve hemostasis/sealing. The use of more than one patch was mostly due to the necessity to cover an area larger than the size of the patch. A maximum of six patches per patient was used, which is in accordance with the IFU [21].

In our registry, MIS patients achieved hemostasis more quickly than patients undergoing open surgery. However, MIS is preferably performed in cases with a lower severity and probability of intraoperative bleeding, and in addition we did not record bleeding grades [20] before product application. Therefore, it cannot be concluded that the Hemopatch^®^ is more effective in achieving hemostasis in MIS vs. open surgery based solely on the observed shorter TTH. However, our results show that the use of Hemopatch^®^ with MIS is safe and effective. In line with our observations, Ulrich et al. did not find differences in successful hemostasis within two min between open procedures and MIS when using Hemopatch^®^ [16]. Hupe et al. reported only a few bleeding events in patients undergoing partial nephrectomy using Hemopatch^®^. Most of these procedures were performed as MIS [11]. Furthermore, Hemopatch® proved to be feasible and reliable in laparoscopic partial nephrectomy [7] and laparoscopic cholecystectomy [13].

We also addressed the satisfaction of surgeons with Hemopatch^®^; this was mostly rated “excellent” or “good”. In accordance with our observations, Ulrich et al. reported a high satisfaction of surgeons across various disciplines with Hemopatch^®^ in their study [16].

As shown in Table 5, surgeons reported a total of 64 AEs in this registry. However, we did not observe any allergic reactions to Hemopatch^®^, even though one of the main components of Hemopatch^®^ is bovine collagen. This is in accordance with prior observations [10]. We found fewer infections at TASs (1.3%) than have been reported in previous studies with Hemopatch^®^ [10, 14]. However, the rate is consistent with the general rate of surgical site infection of 1.9% [26].

Eleven (1.8%) of the patients in this registry experienced an AE that was probably related to Hemopatch^®^ use, as stated by the investigator and/or sponsor. In a published cardiac surgery study with 170 patients [12], no significant differences in postoperative complications were found between Hemopatch^®^ and a control group (compression with dry or wet gauze or similar).

### Limitations

The Hemopatch^®^ registry is limited by the lack of a control group. Furthermore, bleeding grades, e.g. according to the VIBe Scale (Validated Intraoperative Bleeding Scale) [20], were not documented before application of Hemopatch^®^. The eCRF allowed for free text to be entered in the fields of primary indication for surgery, procedure performed, and target application site. The combined analysis of this information allowed a more detailed analysis in most but not all cases. May only become more realistic, if they could be calculated on the basis of relevant cases. Therefore, parameters (such as leak rates per cohort) may only become more realistic, if they could be calculated on the basis of relevant cases.

Due to its dual properties as a “sealing hemostat” and the fact that bleeding grades were not recorded before product application, it was impossible in some cases to determine whether the main purpose of applying the product was hemostasis or sealing.

### Conclusion

The data presented in this registry indicate that the clinical use of Hemopatch^®^ is effective and safe. We showed the effectiveness of Hemopatch^®^ in achieving hemostasis and sealing across various specialties, not only with open surgery approaches but also with MIS. Furthermore, 64.6% of the surgeons rated Hemopatch^®^ as “much better” or “better” than other hemostats, supporting the favorable benefit-to-risk profile of Hemopatch^®^.

### Electronic supplementary material

Below is the link to the electronic supplementary material.

Supplementary file1 (PDF 660 kb)

## Data Availability

The datasets generated during the current study are available from the corresponding author on reasonable request.

## Appendix

Surgeons contributing cases to this registry

Abano Terme, Italy: Daniele D'agostino, Angelo Porreca.

Barcelona, Spain: Itxarone Bilbao Aguirre, Ramon Charco Torra.

Bergisch Gladbach, Germany: Andreas Kohnen, Stefan Machtens.

Berlin, Germany: Petar Petrov, Apostolidis Athanasios, Jaime-Jürgen Eulert-Grehn, MD, Evgenij Potapov, *Mario Zacharias*.

Brno, Czech Republic: Radim Jančálek, Peter Solar.

Castel Volturno (CE), Italy: Rachele Tarquini, Paolo Bianco.

Catania, Italy: Marco Mudanò, *Domenico Russello*, Antonino Scolaro.

Firenze, Italy: Luca Moraldi, Mario Annecchiarico Claudia Paolini.

Girona, Spain: Ernest Castro Gutierrez, *Santiago Lopez-Ben*, Margarida Casella Robert, Laia Falgueras Verdaguer, Maria Teresa Albiol Quer, Fernando Sebastián.

Hamburg, Germany: Nicolai Bayer, Michael Schmoeckel.

Innsbruck, Austria: Reinhold Kafka-Ritsch, Irmgard Kronberger, Manuel Maglione, Rupert Oberhuber, Alexander Perathoner, Stefan Stättner, Florian Primavesi.

Liberec, Czech Republic: Martin Chrenko, Peter Hromadka, Jiri Skach.

Lublin, Poland: Robert Sitarz, Pawel Terlecki, Piotr Gawlik, Marcin Kubiak, Marek Łokaj, Żebrowski Remigiusz, Wiesław Witczymiszyn, Robert Zymon.

Madrid, Spain: Jose Asencio, Ramon Corripio, Irene Osorio Silla, *Elisa York*.

Napoli, Italy: Chiara Offi, Renato Patrone, Roberto Romano, Giovanni Conzo.

Ostrava-Poruba, Czech Republic: *Tomas Hrbac*, *Lukas Krska*, Silvia Szathmáryova.

Pisa, Italy: *Ugo Boggi*, *Carlo Lombardo*, Francesca Menonna, Uberto Bortolotti, Giosue Falcetta.

Prague, Czech Republic: Tomas Vidim, Mirolslav Levy, Marek Mracek.

Regensburg, Germany: Michael Ried, Matthaeus Zerdzitzki, Andreas Holzamer, Michael Hilker, Prof, MD, Lukasz Kmiec, Tobias Potzger.

Sevilla, Spain: Juan Martos, Marta Gonzalez Pombo, *Javier Marquez-Rivas*.

Szczecin, Poland: *Piotr Gutowski*, Maciej Nowacki, Jaroslaw Pawel Szumilowicz.

## Abbreviations

AE: Adverse event
AESI: Adverse event of special interest
CCI: Charlson Comorbidity Index
CI: Confidence interval
CSF: Cerebrospinal fluid
eCRF: Electronic case report form
FAS: Full analysis set
FU: Follow-up
IEC: Independent ethics committee
IFU: Instructions for use
MIS: Minimally invasive surgery
n: Sample size
NHS-PEG: Pentaerythritol poly(ethylene glycol) ether tetra-succinimidyl glutarate
POPF: Postoperative pancreatic fistula
SAS: Safety analysis set
SD: Standard deviation
TAS: Target application site
TTH: Time to hemostasis
