# Supplementary material for: Hemopatch® is effective and safe to use: real-world data from a prospective European registry study
Source: Updates Surg. 2022 Aug 20;74(5):1521–31. doi: 10.1007/s13304-022-01353-y (PMC9481486; doi:10.1007/s13304-022-01353-y)
Supplement: Supplementary file 1 — Supplementary file1 (PDF 660 kb) [file 13304_2022_1353_MOESM1_ESM.pdf]

Baxter: BXU011443 - HEMOPATCH

Annotated CRFs *[Individual forms only displayed once]*

HEMOPATCH PERFORMANCE EVALUATION: A PROSPECTIVE OBSERVATIONAL REGISTRY

Protocol Name: *BXU011443*

XC 14

List of Contents

1. File Information
2. Statistical Information
3. List of Visits and Forms
4. Visit Matrix
5. Case Report Forms
6. New, redefined or unused Definitions
7. Visit Tree

Legend:

*	= Mandatory entry
[#Rep]	= Can be entered repeatedly if necessary
∇	= Will be displayed as drop down in MARVIN
O	= Will be displayed as radio button in MARVIN
☑	= Will be displayed as checked checkbox in MARVIN
☐	= Will be displayed as unchecked checkbox in MARVIN

File Information:

Study OID:	*BHHOBSRG-OBSREG*
File Type:	*Snapshot*
Granularity:	*Metadata*
File OID:	*Export.214401349233958095*
Creation Date and Time:	*2018-04-04T20:31:34+00:00*
As of:	
Date of printout:	*2018-04-13T15:09:21+02:00*
ODM Version:	*1.3*

Metadata Version Information:

Metadata Version OID:	*4.0*
Metadata Version Name:	*4.0*
Metadata Version Description:	*4.0*

Signature Information:

Signature OID:	*sig_01*
Meaning:	*Please be aware that your signature is legally binding.*
Legal Reason:	*Please be aware that your signature is legally binding.*

Statistics

	Definitions	References
Number of Protocols:	1	
Number of Admin Protocols:	2	
Number of Measurement Units:	14	14
Number of Events:	16	16
Number of Forms:	43	78
Number of Item Groups:	48	89
Number of Items:	243	232
Number of Codelists:	43	117
Number of Actions:	186	175

List of Visits and Forms

Subject Protocol

- Registration
  - Eligibility Criteria
  - Subject ID
- Enrollment
  - Subject ID
  - Informed Consent
  - Demographics
  - Relevant Medical History
  - Surgery Information
  - Indication Cohort
- Hospital Stay
  - Subject ID
  - Hospital Stay: {0} - {1}
- Adverse Events and Product Complaints
  - Subject ID
  - Any Adverse Events?
  - Event #{1}: {2} - {0}
  - Product Complaints
  - Product Complaint #{0}: {1}
- Concomitant Medications
  - Subject ID
  - Any relevant Concomitant Medications?
  - Medication #{0}: {1}
- Intra-operative and Post-operative Transfusions
  - Subject ID
  - Transfusions
- Post-operative Surgical Revisions/Re-operations
  - Subject ID
  - Post-operative Surgical Revisions/Re-operations
- Hepatobiliary Surgery: {0}
  - HEMOPATCH Indication Cohort ID
  - Intra-operative Assessment: Hepatobiliary Surgery
  - Bicarbonate Use
  - Post-operative Follow-up Week 1 - {0}
  - Post-operative Follow-up Week 2 - {0}
  - Post-operative Follow-up Week 3 - {0}
  - Post-operative Follow-up Week 4 - {0}
  - Post-operative Follow-up: Hepatobiliary Surgery
- General Surgery: {0}
  - HEMOPATCH Indication Cohort ID
  - Intra-operative Assessment: General Surgery
  - Bicarbonate Use

Post-operative Follow-up Week 1 - {0}
Post-operative Follow-up Week 2 - {0}
Post-operative Follow-up Week 3 - {0}
Post-operative Follow-up Week 4 - {0}
Post-operative Follow-up: General Surgery

Thoracic/Lung Surgery: {0}
HEMOPATCH Indication Cohort ID
Intra-operative Assessment: Thoracic/Lung Surgery
Bicarbonate Use
Post-operative Follow-up Week 1 - {0}
Post-operative Follow-up Week 2 - {0}
Post-operative Follow-up Week 3 - {0}
Post-operative Follow-up Week 4 - {0}
Post-operative Follow-up: Thoracic/Lung Surgery

Cardiovascular Surgery: {0}
HEMOPATCH Indication Cohort ID
Intra-operative Assessment: Cardiovascular Surgery
Bicarbonate Use
Post-operative Follow-up Week 1 - {0}
Post-operative Follow-up Week 2 - {0}
Post-operative Follow-up Week 3 - {0}
Post-operative Follow-up Week 4 - {0}
Post-operative Assessments: Cardiovascular Surgery

Neurological/Spinal Surgery: {0}
HEMOPATCH Indication Cohort ID
Intra-operative Assessment: Neurological/Spinal Surgery
Bicarbonate Use
Post-operative Follow-up Week 1 - {0}
Post-operative Follow-up Week 2 - {0}
Post-operative Follow-up Week 3 - {0}
Post-operative Follow-up Week 4 - {0}
Post-operative Follow-up: Neurological Surgery

Urological Surgery: {0}
HEMOPATCH Indication Cohort ID
Intra-operative Assessment: Urological Surgery
Bicarbonate Use
Post-operative Follow-up Week 1 - {0}
Post-operative Follow-up Week 2 - {0}
Post-operative Follow-up Week 3 - {0}
Post-operative Follow-up Week 4 - {0}
Post-operative Follow-up: Urological Surgery

End of Study
Subject ID
Disposition - End of Study

Administrative Protocol *Medical Center Characteristics*

Medical Center Characteristics

Medical Center Characteristics

Administrative Protocol

HEMOPATCH Questionnaire

HEMOPATCH Intraoperative Use Evaluation Questionnaire
- HEMOPATCH Questionnaire Surgeon 1: {0} {1}
- HEMOPATCH Questionnaire Surgeon 2: {0} {1}
- HEMOPATCH Questionnaire Surgeon 3: {0} {1}
- HEMOPATCH Questionnaire Surgeon 4: {0} {1}
- HEMOPATCH Questionnaire Surgeon 5: {0} {1}

Visit Matrix

orm / vent	Registration	Enrollment	Hospital Stay	Adverse Events and Product Complaints	Concomitant Medications	Intra-operative and Post-operative Transfusions	Post-operative Surgical Revisions/Re-operations	Hepatobiliary Surgery: {0}	General Surgery: {0}	Thoracic/Lung Surgery: {0}	Cardiovascular Surgery: {0}	Neurological/Spinal Surgery: {0}	Urologica Surgery {0}
d Consent		x*											
y Criteria	x*												
ID	x*												
aphics		x*											
ion		x*											
Stay: {0} -			r*										
erative nent: iliary								x*					
erative nent: Surgery									x*				
erative nent: c/Lung										x*			
erative nent: ascular											x*		
erative nent: gical/Spinal												x*	
erative nent: al Surgery													x*
erative s/Re- ns							x*						
ATCH n Cohort								x*	x*	x*	x*	x*	x*
1}: {2} - {0}				r*									
sions						x*							
erative p: iliary								x*					
ion - End													
erative p: General									x*				
t Medical		x*											
n Cohort		x*											
erative p: c/Lung										x*			
erative p: gical												x*	
erative p: al Surgery													x*
on #{0}:					r*								

erse			x*									
vant itant ons?				x*								
ID	x*	x*	x*	x*	x*	x*						
erative nents: ascular										x*		
Center eristics												
erative p Week 1 -							x*	x*	x*	x*	x*	x*
erative p Week 2 -							x*	x*	x*	x*	x*	x*
erative p Week 3 -							x*	x*	x*	x*	x*	x*
erative p Week 4 -							x*	x*	x*	x*	x*	x*
nts			x*									
nate Use							x*	x*	x*	x*	x*	x*
ATCH nnaire 2: {0} {1}												
ATCH nnaire 3: {0} {1}												
ATCH nnaire 4: {0} {1}												
ATCH nnaire 5: {0} {1}												
ATCH nnaire 1: {0} {1}												
Complaint			r*									

Subject Protocol

Registration * *e_REGISTER; Scheduled*

Eligibility Criteria* *f_ieel*

Patient must satisfy ALL of the following inclusion criteria before they will be allowed to participate in the registry.

Inclusion Criteria* *g_incl; SAS:INCL*

Inclusion #1: Male or female patient of any age* *i_incl1; text1; SAS:INCL1*	∇ Y Yes ∇ N No *c_ny; text; SAS:$NY; display.style radio* *ARC arc_default*
Inclusion #2: Patient has undergone open or minimal invasive surgical (MIS) procedures requiring the use of a patch to achieve dura repair, hemostasis, or control of leakage of air or other body fluid when pressure, ligature or other conventional procedures had been ineffective or impractical and where use of HEMOPATCH was deemed appropriate by the surgeon according to its IFU in at least one of the organ systems specified in protocol.* *i_incl2; text1; SAS:INCL2*	∇ Y Yes ∇ N No *c_ny; text; SAS:$NY; display.style radio* *ARC arc_default*
Inclusion #3: Provision of written informed consent/assent, as applicable, up to 72 hours after the date of surgery.* *i_incl3; text1; SAS:INCL3*	∇ Y Yes ∇ N No *c_ny; text; SAS:$NY; display.style radio* *ARC arc_default*

Patients fulfilling ANY of the following exclusion criteria will not be allowed to participate in the registry.

Exclusion Criteria* *g_excl; SAS:EXCL*

Exclusion #1: Patient has a known hypersensitivity to bovine proteins or brilliant blue* *i_excl1; text1; SAS:EXCL1*	∇ Y Yes ∇ N No *c_ny; text; SAS:$NY; display.style radio* *ARC arc_default*
Exclusion #2: Patient had intraoperative pulsatile or severe bleeding at the TAS* *i_excl2; text1; SAS:EXCL2*	∇ Y Yes ∇ N No *c_ny; text; SAS:$NY; display.style radio* *ARC arc_default*
Exclusion #3: Patient had an active infection at the TAS* *i_excl3; text1; SAS:EXCL3*	∇ Y Yes ∇ N No *c_ny; text; SAS:$NY; display.style radio* *ARC arc_default*

Subject ID* *f_subj*

Subject ID* *g_subj; SAS:SUBJ*

Site ID* *i_siteid; text6; SAS:SITEID* *CALCULATEONCE the i_siteid when all items within g_incl = Y, and all items within g_excl = N. i_siteid to be populated with 6-character Center # from Center Affix (in center administration details).*	_ _ _ _ _ _ *ARC arc_default*

Subject ID* *g_subj; SAS:SUBJ*

Subject ID* *i_subjid; text11; SAS:SUBJID*<br>*CALCULATEONCE the i_subjid when all items within g_incl = Y, and all items within g_excl = N. Unique Subject ID format (XXYYYY-ZZZZ): Concatenate 6-character i_siteid from the Center details (center affix) + 4-digit site-wide patient counter e.g. CZ0029-0001, CZ0029-0002, CZ0029-0003, etc.*	_ _ _ _ _ _ _ _ _ _ _	*ARC arc_default*

*Enrollment** *e_ENROLLMENT; Scheduled*

CALCULATEEVENT: create e_ENROLLMENT event when i_subjid is assigned by copying i_subjid into i_subjidcopy on the f_subjcopy form within the e_ENROLLMENT event.

Subject ID* *f_subjcopy*

Subject ID* *g_subjcopy; SAS:SUBJCOPY*

Subject ID* *i_subjidcopy; text11; SAS:SUBJCOPY* *NOTE: i_subjid will be copied into i_subjidcopy via calculateEvent to create all non-cohort specific events (e.g. e_HOSP, e_AE, e_CM, e_EOS, etc...).*	_ _ _ _ _ _ _ _ _ _ _ *ARC arc_default*

Informed Consent* *f_dsic*

Informed Consent* *g_dsic; SAS:DSIC*

Date of Informed Consent Signature:* *i_rficdat; date10; SAS:RFICDAT*	_ _ /_ _ /_ _ _ _ mm/dd/yyyy *ARC arc_criticalData*
Informed Consent Version Number *i_rficvers; float2,1; SAS:RFICVERS*	_ , _ *ARC arc_default*
Informed Consent Version Date* *i_rficvdat; date10; SAS:RFICVDAT*	_ _ /_ _ /_ _ _ _ mm/dd/yyyy *ARC arc_default*
Was informed assent obtained?* *i_rfiayn; text3; SAS:RFIAYN*	∇ *Y* Yes ∇ *N* No ∇ *N/A* Not Applicable *c_nyna; text; SAS:$NYNA; display.style radio* *ARC arc_default*
Date of Informed Assent Signature:* *i_rfiadat; date10; SAS:RFIADAT* *ENABLE i_rfiadat if i_rfiayn = Y*	_ _ /_ _ /_ _ _ _ mm/dd/yyyy *ARC arc_criticalData*
Protocol Version* *i_protvers; text100; SAS:PROTVERS* *EXTERNAL CODELIST: will be uploaded externally to study definition, without need for an amendment. Codelist will include Protocol version # and version date (e.g. 1.0 - 22-Jun-2017).*	External Codelist: `c_protvers` *c_protvers; text; SAS:$PROTVER* *External codelist of protocol versions (including # and date, e.g. 1.0 - 22-Jun-2017)* *ARC arc_default*

If re-consent was obtained, please click 'Add ItemGroup' below to record details of each additional consent: [# _ _] *g_dsic2; SAS:DSIC2; display.style horizontal*

Date of Re-consent:* *i_rficdat2; date10; SAS:RFICDAT2*	_ _ /_ _ /_ _ _ _ mm/dd/yyyy *ARC arc_default*
Informed Consent Version Number* *i_rficvs2; float2,1; SAS:RFICVS2*	_ , _ *ARC arc_default*
Informed Consent Version Date* *i_rficvdt2; date10; SAS:RFICVDT2*	_ _ /_ _ /_ _ _ _ mm/dd/yyyy *ARC arc_default*
Protocol Version* *i_protvs2; text100; SAS:PROTVS2* *EXTERNAL CODELIST: will be uploaded externally to study definition. Will include version # and version date (e.g. 1.0 - 22-Jun-2017). List can be updated by CSD as additional protocol versions are relevant.*	External Codelist: `c_protvers` *c_protvers; text; SAS:$PROTVER* *External codelist of protocol versions (including # and date, e.g. 1.0 - 22-Jun-2017)* *ARC arc_default*

Demographics* *f_dm*

Demographics* *g_dm; SAS:DM*

Age at Time of Informed Consent* *i_age; integer3; SAS:AGE*	_ _ _ *ARC arc_default*
Age Unit* *i_ageu; text6; SAS:AGEU*	∇ *MONTHS* Month(s) ∇ *YEARS* Year(s) *c_ageu; text; SAS:$AGEU*<br>*ARC arc_default*
Sex* *i_sex; text16; SAS:SEX*	∇ *M* Male ∇ *F* Female *c_sex; text; SAS:$SEX; display.style radio*<br>*ARC arc_default*
Ethnicity** *i_ethnic; text25; SAS:ETHNIC*	∇ *HISPANIC OR LATINO* Hispanic or Latino ∇ *NOT HISPANIC OR LATINO* Not Hispanic or Latino ∇ *NOT REPORTED* Not Reported ∇ *UNKNOWN* Unknown *c_ethnic; text; SAS:$ETHNIC*<br>*ARC arc_default*
Race** *i_race; text50; SAS:RACE*	∇ *AMERICAN INDIAN OR ALASKA NATIVE* American Indian or Alaska Native ∇ *ASIAN* Asian ∇ *BLACK OR AFRICAN AMERICAN* Black or African American ∇ *NATIVE HAWAIIAN OR OTHER PACIFIC ISLANDER* Native Hawaiian or Other Pacific Islander ∇ *WHITE* White ∇ *OTHER* Other *c_race; text; SAS:$RACE*<br>*ARC arc_default*
If "Other", please specify* *i_raceoth1; text200; SAS:RACEOTH*<br>*ENABLE i_raceoth1 if i_race = OTHER*	_ _<br>_ _<br>_ _<br>_ _<br>_ _<br>_ _<br>_ _<br>_ _<br>_ _ _ _ _ _ _ _ _ _ _ _ _ _ _ _ *ARC arc_default*
Is the subject of child-bearing potential?* *i_cbpyn; text1; SAS:CBPYN*<br>*ENABLE i_cbpyn if i_sex <> M*	∇ *Y* Yes ∇ *N* No *c_ny; text; SAS:$NY; display.style radio*<br>*ARC arc_criticalData*
If the subject is not of child bearing potential, then please provide a reason:* *i_cbpreas; text20; SAS:CBPREAS*<br>**a_e_dm_003 : ENABLE i_cbpreas if i_cbpyn = N*	∇ *SURGICALLY STERILE* Surgically Sterile ∇ *POST-MENOPAUSAL* Post-menopausal ∇ *PREPUBESCENT* Prepubescent ∇ *OTHER* Other *c_cbpreas; text; SAS:$CBPREAS*<br>*ARC arc_default*
If "Other", please specify other reason:* *i_cbpreaso; text200; SAS:CBPREASO*<br>*ENABLE i_cbpreaso if i_cbpreas = OTHER*	_ _<br>_ _<br>_ _<br>_ _<br>_ _<br>_ _<br>_ _<br>_ _<br>_ _ _ _ _ _ _ _ _ _ _ _ _ _ _ _ *ARC arc_default*

*Based on FDA Guidance for industry: Collection of Race and Ethnicity data in clinical trials

Relevant Medical History* *f_mh*

Any relevant medical history?* *g_mhyn; SAS:MHYN*

Does the subject have any relevant medical history indicative of bleeding disorder (i.e. liver disease, inherent coagulopathies, etc.)?* *i_mhyn; text3; SAS:MHYN*	∇Y Yes ∇N No ∇UNK Unknown *c_nyunk; text; SAS:$NYUNK; display.style radio* ARC arc_default

If "Yes", please record all relevant medical history below. Click 'Add ItemGroup' to add additional rows: [# _ _]* *g_mh; SAS:MH; display.style horizontal*
ENABLE g_mh item group if i_mhyn = Y

Medical History #: *i_mhspid; integer2; SAS:MHSPID* *CALCULATEONCE the i_mhspid by counting the # of item groups added to the f_mh form starting at 1.*	_ _ ARC arc_default
Medical History Condition* *i_mhterm; text200; SAS:MHTERM*	_ ARC arc_default
Start Date - Day* *i_mhstdy; text2; SAS:MHSTDY*	∇UN Unknown ∇01 01 ∇02 02 ∇03 03 ∇04 04 ∇05 05 ∇06 06 ∇07 07 ∇08 08 ∇09 09 ∇10 10 ∇11 11 ∇12 12 ∇13 13 ∇14 14 ∇15 15 ∇16 16 ∇17 17 ∇18 18 ∇19 19 ∇20 20 ∇21 21 ∇22 22 ∇23 23 ∇24 24 ∇25 25 ∇26 26 ∇27 27 ∇28 28 ∇29 29 ∇30 30 ∇31 31 *c_caldays; text; SAS:$CALDAY* ARC arc_default
Start Date - Month* *i_mhstmo; text3; SAS:MHSTMO*	∇UNK Unknown ∇JAN Jan ∇FEB Feb ∇MAR Mar ∇APR Apr ∇MAY May ∇JUN Jun ∇JUL Jul ∇AUG Aug ∇SEP Sep ∇OCT Oct ∇NOV Nov ∇DEC Dec *c_calmonu; text; SAS:$CALMONU* ARC arc_default
Start Date - Year* *i_mhstyr; integer4; SAS:MHSTYR*	_ _ _ _ ARC arc_default
Ongoing?* *i_mhongo; text1; SAS:MHONGO*	∇Y Yes ∇N No *c_ny; text; SAS:$NY; display.style radio* ARC arc_default
End Date - Day* *i_mhendy; text2; SAS:MHENDY* *ENABLE i_mhendy, i_mhenmo, i_mhenyr if i_mhongo = N*	∇UN Unknown ∇01 01 ∇02 02 ∇03 03 ∇04 04 ∇05 05 ∇06 06 ∇07 07 ∇08 08 ∇09 09 ∇10 10 ∇11 11 ∇12 12 ∇13 13 ∇14 14 ∇15 15 ∇16 16 ∇17 17 ∇18 18 ∇19 19 ∇20 20 ∇21 21 ∇22 22 ∇23 23 ∇24 24 ∇25 25 ∇26 26 ∇27 27 ∇28 28 ∇29 29 ∇30 30 ∇31 31 *c_caldays; text; SAS:$CALDAY* ARC arc_default
End Date - Month* *i_mhenmo; text3; SAS:MHENMO* *ENABLE i_mhendy, i_mhenmo, i_mhenyr if i_mhongo = N*	∇UNK Unknown ∇JAN Jan ∇FEB Feb ∇MAR Mar ∇APR Apr ∇MAY May ∇JUN Jun ∇JUL Jul ∇AUG Aug ∇SEP Sep ∇OCT Oct ∇NOV Nov ∇DEC Dec *c_calmonu; text; SAS:$CALMONU* ARC arc_default
End Date - Year* *i_mhenyr; integer4; SAS:MHENYR* *ENABLE i_mhendy, i_mhenmo, i_mhenyr if i_mhongo = N*	_ _ _ _ ARC arc_default

Surgery Information* *f_surgery*

Surgery Information* *g_pr; SAS:PR*

Surgeon First Name:* *i_sgnam1; text20; SAS:SGNAM1*	_ *ARC arc_default*
Surgeon Last Name:* *i_sgnam2; text20; SAS:SGNAM2*	_ *ARC arc_default*
Surgeon Title:* *i_sgnam3; text20; SAS:SGNAM3*	_ *ARC arc_default*
Procedure Description* *i_prterm; text200; SAS:PRTERM*	_ *ARC arc_default*
Primary Indication* *i_prindc; text200; SAS:PRINDC*	_ *ARC arc_default*
Surgical Approach* *i_prapprch; text20; SAS:PRAPPRCH*	∇ *OPEN* Open Surgery ∇ *MIS* Minimally Invasive Surgery (MIS) *c_prapp; text; SAS:$PRAPP* *ARC arc_default*
Surgery Start Date* *i_prstdat; date10; SAS:PRSTDAT*	_ _ /_ _ /_ _ _ _ mm/dd/yyyy *ARC arc_default*
Surgery Start Time* *i_prsttim; time5; SAS:PRSTTIM*	_ _ :_ _ hh:mm *ARC arc_default*
Surgery End Date* *i_prendat; date10; SAS:PRENDAT*	_ _ /_ _ /_ _ _ _ mm/dd/yyyy *ARC arc_default*
Surgery End Time* *i_prentim; time5; SAS:PRENTIM*	_ _ :_ _ hh:mm *ARC arc_default*

Comorbidities *g_cci; SAS:CCI*

Does the subject have any comorbidities?* *i_prcciyn; text3; SAS:PRCCIYN*	∇ *Y* Yes ∇ *N* No ∇ *UNK* Unknown *c_nyunk; text; SAS:$NYUNK; display.style radio* *ARC arc_default*

Comorbidities *g_cci; SAS:CCI*

If "Yes", please provide Charlson Comorbidity Index (CCI) Total Score:* *i_prcci; integer2; SAS:PRCCI* __ __ *ARC arc_default*

ENABLE i_prcci if i_prcciyn = Y

Online Calculator:
https://www.mdcalc.com/charlson-comorbidity-index-cci

Guide for Calculating CCI Total Score:

Age (

Age (50-59 years) +1

Age (60-69 years) +2

Age (70-79) years +3

Age (≥80 years)+4

Diabetes mellitus (Uncomplicated) +1

Diabetes mellitus (End-organ damage) +2

Liver disease (Mild) +1

Liver disease (Moderate to Severe) +3

Solid tumor (Localized) +2

Solid tumor (Metastatic) +6

AIDS +6

Moderate to severe CKD +2

CHF +1

Myocardial infarction +1

COPD +1

Peripheral vascular disease +1

CVA or TIA +1

Dementia +1

Hemiplegia +2

Connective tissue disease +1

[illegible]

Indication Cohort* *f_cohort*

HEMOPATCH Indication Cohort (Check all that apply)* *g_cohort; SAS:COHORT*

Indication Cohort: Hepatobiliary Surgery *i_coliver; text1; SAS:COLIVER; display.style checkbox*	☑ *Y c_checkyes; text; SAS:$CHKYES; display.style checkbox* *ARC arc_default*
Indication Cohort: General Surgery *i_cogeneral; text1; SAS:COGENRL; display.style checkbox*	☑ *Y c_checkyes; text; SAS:$CHKYES; display.style checkbox* *ARC arc_default*
Indication Cohort: Lung/Thoracic Surgery *i_colung; text1; SAS:COLUNG*	☑ *Y c_checkyes; text; SAS:$CHKYES; display.style checkbox* *ARC arc_default*
Indication Cohort: Cardiovascular Surgery *i_cocardio; text1; SAS:COCARDIO*	☑ *Y c_checkyes; text; SAS:$CHKYES; display.style checkbox* *ARC arc_default*
Indication Cohort: Neurological/Spinal Surgery *i_coneuro; text1; SAS:CONEURO*	☑ *Y c_checkyes; text; SAS:$CHKYES; display.style checkbox* *ARC arc_default*
Indication Cohort: Urological Surgery *i_courolog; text1; SAS:COUROLOG*	☑ *Y c_checkyes; text; SAS:$CHKYES; display.style checkbox* *ARC arc_default*
Cohort ID - Hepatobiliary Surgery (Derived) *i_colivid; text10; SAS:COLIVID* *CALCULATE ONCE to derive cohort ID. Will be used to trigger e_LIVER event via CalculateEvent.*	_ _ _ _ _ _ _ _ _ _ *ARC arc_default*
Cohort ID - General Surgery (Derived) *i_cogenid; text10; SAS:COGENID* *CALCULATEONCE to derive cohort ID. Will be used to trigger e_GENERAL event via CalculateEvent.*	_ _ _ _ _ _ _ _ _ _ *ARC arc_default*
Cohort ID - Thoracic/Lung Surgery (Derived) *i_colungid; text10; SAS:COLUNGID* *CALCULATEONCE to derive cohort ID. Will be used to trigger e_LUNG event via CalculateEvent.*	_ _ _ _ _ _ _ _ _ _ *ARC arc_default*
Cohort ID - Cardiovascular Surgery (Derived) *i_cocardid; text10; SAS:COCARDID* *CALCULATEONCE to derive cohort ID. Will be used to trigger e_CARDIO event via CalculateEvent.*	_ _ _ _ _ _ _ _ _ _ *ARC arc_default*
Cohort ID - Neurological/Spinal Surgery (Derived) *i_coneurid; text10; SAS:CONEURID* *CALCULATEONCE to derive cohort ID. Will be used to trigger e_NEURO event via CalculateEvent.*	_ _ _ _ _ _ _ _ _ _ *ARC arc_default*
Cohort ID - Urological Surgery (Derived) *i_courolid; text10; SAS:COUROLID* *CALCULATEONCE to derive cohort ID. Will be used to trigger e_UROLOGIC event via CalculateEvent.*	_ _ _ _ _ _ _ _ _ _ *ARC arc_default*
List of All Cohort IDs for this Subject (Derived): *i_coidall; text60; SAS:COIDALL* *CALCULATEONCE - concatenate the following: i_colivid, i_cogenid, i_colungid, i_cocardid, i_coneurid, i_courolid. Separate with ",". For displaying in Base Data info.*	_ *ARC arc_default*

*Hospital Stay** *e_HOSP; Common*

CALCULATEEVENT: create e_HOSP event when i_subjid is assigned by copying i_subjid into i_subjidcopy on the f_subjcopy form within the e_HOSP event.

Subject ID* *f_subjcopy*

- [Form already referred to in a previous event!]

Hospital Stay: {0} - {1} [# _ _]* *f_hosp; EP(0): ItemGroupData[@OID="g_hosp"]/ItemData[@OID="i_hospstdat"]; EP(1): ItemGroupData[@OID="g_hosp"]/ItemData[@OID="i_hospendat"]*

Hospital Admittance/Discharge* *g_hosp; SAS:HOSP*

Date Admitted into Hospital* *i_hospstdat; date10; SAS:HOSPSTDT*	_ _ /_ _ /_ _ _ _ mm/dd/yyyy *ARC arc_default*
Date Discharged from Hospital* *i_hospendat; date10; SAS:HOSPENDT*	_ _ /_ _ /_ _ _ _ mm/dd/yyyy *ARC arc_default*
Was the subject admitted into the ICU during this hospital stay?* *i_hospicuyn; text1; SAS:ICUYN*	∇ Y Yes ∇ N No *c_ny; text; SAS:$NY; display.style radio* *ARC arc_default*

If "Yes", please record time in ICU below. Click 'Add ItemGroup' to record additional stays in ICU during this hospitalization, if applicable. [# _ _]* *g_icu; SAS:ICU; display.style horizontal*

ENABLE item group g_icu if i_hospicuyn = Y

Date Admitted into ICU* *i_icustdat; date10; SAS:ICUSTDAT*	_ _ /_ _ /_ _ _ _ mm/dd/yyyy *ARC arc_default*
Time Admitted into ICU* *i_icusttim; time5; SAS:ICUSTTIM*	_ _ :_ _ hh:mm *ARC arc_default*
Date Discharged from ICU* *i_icuendat; date10; SAS:ICUENDAT*	_ _ /_ _ /_ _ _ _ mm/dd/yyyy *ARC arc_default*
Time Discharged from ICU* *i_icuentim; time5; SAS:ICUENTIM*	_ _ :_ _ hh:mm *ARC arc_default*

If the subject returns to the hospital after initial discharge due to complications, re-operations, etc., add a new Hospital Stay form to record additional hospital stays.

Adverse Events and Product Complaints *e_AE; Scheduled

CALCULATEEVENT: create e_AE event when i_subjid is assigned by copying i_subjid into i_subjidcopy on the f_subjcopy form within the e_AE event.

Subject ID* *f_subjcopy*

- [Form already referred to in a previous event!]

Any Adverse Events?* *f_aeyn*

Documentation of Serious Adverse Events, Adverse Events of Special Interest, and Adverse Device Events (from the time of Enrollment up to 4 weeks post-surgery)* *g_aeyn; SAS:AEYN*

Did the subject experience any serious adverse events?* *i_aeynsae; text3; SAS:AEYNSAE*	∇Y Yes ∇N No ∇UNK Unknown *c_nyunk; text; SAS:$NYUNK; display.style radio* ARC arc_default
Did the subject experience any adverse events of special interest (AESI)?* *i_aeynaesi; text3; SAS:AEYNAESI*	∇Y Yes ∇N No ∇UNK Unknown *c_nyunk; text; SAS:$NYUNK; display.style radio* ARC arc_default
Did the subject experience any adverse events that were determined to be related to HEMOPATCH?* *i_aeynade; text3; SAS:AEYNADE*	∇Y Yes ∇N No ∇UNK Unknown *c_nyunk; text; SAS:$NYUNK; display.style radio* ARC arc_default

If "Yes" is answered to any of the above questions, please add an Adverse Event form (in the left side panel) to record each relevant event.

Event #{1}: {2} - {0} [# _ _]* *f_ae; EP(0): ItemGroupData[@OID="g_ae"]/ItemData[@OID="i_aestdat"]; EP(1): ItemGroupData[@OID="g_ae"]/ItemData[@OID="i_aespid"]; EP(2): ItemGroupData[@OID="g_ae"]/ItemData[@OID="i_aeterm"]*

ENABLE f_ae if (i_aeynsae = Y or i_aeynaesi = Y or i_aeynade = Y)

Documentation of Serious Adverse Event (SAE), Adverse Event of Special Interest (AESI), or Adverse Device Event (ADE)* *g_ae; SAS:AE*

Adverse Event #:* *i_aespid; integer2; SAS:AESPID* *CALCULATEONCE i_aespid by counting f_ae forms within the patient, starting at 1.*	_ _ ARC arc_default
Adverse Event* *i_aeterm; text200; SAS:AETERM*	_ (10 lines) ARC arc_default
Is the event a Serious Adverse Event (SAE)?* *i_aeser; text1; SAS:AESER; display.style radio*	OY Yes ON No *c_ny; text; SAS:$NY; display.style radio* ARC arc_default

Documentation of Serious Adverse Event (SAE), Adverse Event of Special Interest (AESI), or Adverse Device Event (ADE)* *g_ae; SAS:AE*

Is the event an Adverse Event of Special Interest (AESI)?* *i_aesi; text1; SAS:AESI*	∇ Y Yes ∇ N No *c_ny; text; SAS:$NY; display.style radio* ARC arc_default
Start Date* *i_aestdat; date10; SAS:AESTDAT*	_ _ /_ _ /_ _ _ _ mm/dd/yyyy ARC arc_default
Approximate Start Time* *i_aesttim; time5; SAS:AESTTIM*	_ _ :_ _ hh:mm ARC arc_default
Ongoing?* *i_aeongo; text1; SAS:AEONGO; display.style radio*	O Y Yes O N No *c_ny; text; SAS:$NY; display.style radio* ARC arc_default
End Date* *i_aeendat; date10; SAS:AEENDAT* *ENABLE i_aeendat and i_aeentim if i_aeongo = N*	_ _ /_ _ /_ _ _ _ mm/dd/yyyy ARC arc_default
Approximate End Time* *i_aeentim; time5; SAS:AEENTIM* *ENABLE i_aeendat and i_aeentim if i_aeongo = N*	_ _ :_ _ hh:mm ARC arc_default
Severity* *i_aesev; text8; SAS:AESEV*	∇ MILD Mild ∇ MODERATE Moderate ∇ SEVERE Severe *c_aesev; text; SAS:$AESEV; display.style radio.vertical* ARC arc_default
Relationship to HEMOPATCH* *i_aerel; text25; SAS:AEREL*	∇ NOT RELATED Not Related ∇ UNLIKELY RELATED Unlikely Related ∇ POSSIBLY RELATED Possibly Related ∇ PROBABLY RELATED Probably Related *c_aerel; text; SAS:$AEREL* ARC arc_default
Relationship to Study Procedure* *i_aeprrel; text20; SAS:AEPRREL*	∇ NOT RELATED Not Related ∇ UNLIKELY RELATED Unlikely Related ∇ POSSIBLY RELATED Possibly Related ∇ PROBABLY RELATED Probably Related *c_aerel; text; SAS:$AEREL* ARC arc_default
If event is "Not Related" or "Unlikely Related" to HEMOPATCH or study procedure, please provide alternate causality:* *i_aereloth; text200; SAS:AERELOTH* *ENABLE i_aereloth if [i_aerel = (NOT RELATED or UNLIKELY RELATED) and if i_aeprrel = (NOT RELATED or UNLIKELY RELATED)]*	_ ARC arc_default
Action Taken with HEMOPATCH *i_aeacn; text200; SAS:AEACN*	∇ NONE None ∇ HEMOPATCH UNIT REPLACED HEMOPATCH Unit Replaced ∇ INCREASED HEMOPATCH UNITS Increased Number of HEMOPATCH Units ∇ HEMOPATCH WITHDRAWN HEMOPATCH Withdrawn ∇ UNKNOWN Unknown ∇ OTHER Other *c_aeacn; text; SAS:$AEACN* ARC arc_default
If "Other", please specify:* *i_aeacnoth; text200; SAS:AEACNOTH* *ENABLE i_aeacnoth if i_aeacn = OTHER*	_ ARC arc_default

Documentation of Serious Adverse Event (SAE), Adverse Event of Special Interest (AESI), or Adverse Device Event (ADE)* *g_ae; SAS:AE*

Outcome* *i_aeout; text32; SAS:AEOUT*	∇ *RECOVERING/RESOLVING* Recovering/Resolving ∇ *RECOVERED/RESOLVED WITH SEQUELAE* Recovered/Resolved with Sequelae ∇ *RECOVERED/RESOLVED* Recovered/Resolved ∇ *NOT RECOVERED/NOT RESOLVED* Not Recovered/Not Resolved ∇ *FATAL* Fatal ∇ *UNKNOWN* Unknown *c_aeout; text; SAS:$AEOUT* *ARC arc_default*

Serious Adverse Event Details* *g_sae; SAS:SAESUPP*

ENABLE g_sae item group if i_aeser = Y

Did the serious event result in persistent or significant disability/incapacity?* *i_aesdisab; text1; SAS:AESDISAB; display.style radio*	O *Y* Yes O *N* No *c_ny; text; SAS:$NY; display.style radio* *ARC arc_default*
Was the serious event associated with congenital anomaly or birth defect?* *i_aescong; text1; SAS:AESCONG; display.style radio*	O *Y* Yes O *N* No *c_ny; text; SAS:$NY; display.style radio* *ARC arc_default*
Did the serious event result in death?* *i_aesdth; text1; SAS:AESDTH; display.style radio*	O *Y* Yes O *N* No *c_ny; text; SAS:$NY; display.style radio* *ARC arc_default*
Did the serious event require or prolong hospitalization?* *i_aeshosp; text1; SAS:AESHOSP; display.style radio*	O *Y* Yes O *N* No *c_ny; text; SAS:$NY; display.style radio* *ARC arc_default*
Was the serious event life threatening?* *i_aeslife; text1; SAS:AESLIFE; display.style radio*	O *Y* Yes O *N* No *c_ny; text; SAS:$NY; display.style radio* *ARC arc_default*
Was the adverse event a medically important event not covered by other serious criteria?* *i_aesmie; text1; SAS:AESMIE; display.style radio*	O *Y* Yes O *N* No *c_ny; text; SAS:$NY; display.style radio* *ARC arc_default*

Product Complaints* *f_pc*

Product Complaints* *g_pcyn; SAS:PCYN*

Are there any HEMOPATCH product complaints to report that are associated with this subject?* *i_pcyn; text1; SAS:PCYN*	∇ *Y* Yes ∇ *N* No *c_ny; text; SAS:$NY; display.style radio* *ARC arc_default*

Product Complaint #{0}: {1} [# _ _]* *f_2pc; EP(0): ItemGroupData[@OID="g_2pc"]/ItemData[@OID="i_pcspid"]; EP(1): ItemGroupData[@OID="g_2pc"]/ItemData[@OID="i_pcserial"]*

Please provide the details for the product complaints below:* *g_2pc; SAS:P2C*

Update: ENABLE f_2pc if i_pcyn = Y

Product Complaint #: *i_pcspid; integer2; SAS:PCSPID* *CALCULATEONCE the i_pcspid by counting g_pc item groups within the f_pc form for this patient, starting at 1*	_ _ *ARC arc_default*

Please provide the details for the product complaints below:* *g_2pc; SAS:P2C*

Update: ENABLE f_2pc if i_pcyn = Y

Product Complaint Description* *i_pcterm; text200; SAS:PCTERM*	_ _ _ _ _ _ _ _ _ _ _ _ _ _ _ _ _ _ _ _ _ _ _ _ *ARC arc_default*
Date Event Occurred *i_pcdat; date10; SAS:PCDAT*	_ _ /_ _ /_ _ _ _ mm/dd/yyyy *ARC arc_default*
If Date Event Occurred is not completed, please provide: *i_pcdatnd; text50; SAS:PCDATND*	∇ *REPORTER DID NOT KNOW* Reporter Did Not Know ∇ *NOT ASKED* Not Asked *c_p1c; text; SAS:$P1C; display.style radio* *ARC arc_default*
Please provide the **Product Code, Serial Number or Lot Number** of the HEMOPATCH complaint:* *i_pcserial; text20; SAS:PCSERIAL*	_ *ARC arc_default*
Is this complaint related to a reported Adverse Event?* *i_pcaerel; text1; SAS:PCAEREL*	∇ *Y* Yes ∇ *N* No *c_ny; text; SAS:$NY; display.style radio* *ARC arc_default*
Please provide corresponding AE number:* *i_pcae1no; text3; SAS:PCAE1NO* *Enable i_pcae1no, i_pcae2no, i_pcae3no, i_pcae4no, i_pcae5no, i_pcae6no if i_pcaerel = Y*	_ _ _ *ARC arc_default*
**If there are additional corresponding AE(s), please enter each AE number on each separate line.** Additional corresponding AE number (if applicable): *i_pcae2no; text3; SAS:PCAE2NO*	_ _ _ *ARC arc_default*
Additional corresponding AE number (if applicable): *i_pcae3no; text3; SAS:PCAE3NO*	_ _ _ *ARC arc_default*
Additional corresponding AE number (if applicable): *i_pcae4no; text3; SAS:PCAE4NO*	_ _ _ *ARC arc_default*
Additional corresponding AE number (if applicable): *i_pcae5no; text3; SAS:PCAE5NO*	_ _ _ *ARC arc_default*
Additional corresponding AE number (if applicable): *i_pcae6no; text3; SAS:PCAE6NO*	_ _ _ *ARC arc_default*
**PATIENT INFORMATION** Did the event occur during patient use or during set up for patient use?* *i_pcptuse; text50; SAS:PCPTUSE*	∇ *PATIENT USE* Patient Use ∇ *PATIENT SET UP* Patient Set Up ∇ *REPORT DID NOT KNOW* Reporter Did Not Know ∇ *NO* No ∇ *NOT ASKED* Not Asked *c_p4c; text; SAS:$P4C* *ARC arc_default*
Did the patient receive treatment as a result of this event?* *i_pctxt; text50; SAS:PCTXT*	∇ *Y* Yes ∇ *N* No ∇ *REPORTER DID NOT KNOW* Reporter Did Not Know ∇ *NOT ASKED* Not Asked *c_p6c; text; SAS:$P6C* *ARC arc_default*

Please provide the details for the product complaints below:* *g_2pc; SAS:P2C*
Update: ENABLE f_2pc if i_pcyn = Y

If Yes, please specify the treatment: **Please provide the general treatment (i.e. medications) and any specifications can be recorded on the corresponding CRF (i.e. Concomitant Medications). *** *i_pctxtsp; text200; SAS:PCTXTSP* *Enable i_pctxtsp if i_pctxt = Y*	________________________________ ________________________________ ________________________________ ________________________________ ________________________________ ________________________________ ________________________________ ________________________________ ______________________ *ARC arc_default*

*Concomitant Medications** *e_CM; Scheduled*

CALCULATEEVENT: create e_CM event when i_subjid is assigned by copying i_subjid into i_subjidcopy on the f_subjcopy form within the e_CM event.

Subject ID* *f_subjcopy*

- [Form already referred to in a previous event!]

Any relevant Concomitant Medications?* *f_cmyn*

Relevant Concomitant Medications* *g_cmyn; SAS:CMYN*

Is the subject taking any anti-coagulant or antiplatelet treatment?* *i_cmsiyn; text1; SAS:CMSIYN*	∇ *Y* Yes ∇ *N* No *c_ny; text; SAS:$NY; display.style radio* *ARC arc_default*

If "Yes", please add a Concomitant Medications form (in the left side panel) to record each relevant medication.

Medication #{0}: {1} [# _ _]* *f_cm; EP(0): ItemGroupData[@OID="g_cm"]/ItemData[@OID="i_cmspid"]; EP(1): ItemGroupData[@OID="g_cm"]/ItemData[@OID="i_cmtrt"]*

ENABLE f_cm form if i_cmyn = Y

Concomitant Medication* *g_cm; SAS:CM*

Medication #:* *i_cmspid; integer2; SAS:CMSPID* *CALCULATEONCE i_cmspid by counting f_cm forms within the patient, starting at 1.*	_ _ *ARC arc_default*
Medication (Report Generic Name whenever possible)* *i_cmtrt; text200; SAS:CMTRT*	_ (×9 lines) _ _ _ _ _ _ _ _ _ _ _ _ _ _ _ _ *ARC arc_default*

Concomitant Medication* g_cm; SAS:CM

Indication* i_cmindc; text200; SAS:CMINDC	______________________________ ARC arc_default
Start Date - Day* i_cmstdy; text2; SAS:CMSTDY	∇ UN Unknown ∇ 01 01 ∇ 02 02 ∇ 03 03 ∇ 04 04 ∇ 05 05 ∇ 06 06 ∇ 07 07 ∇ 08 08 ∇ 09 09 ∇ 10 10 ∇ 11 11 ∇ 12 12 ∇ 13 13 ∇ 14 14 ∇ 15 15 ∇ 16 16 ∇ 17 17 ∇ 18 18 ∇ 19 19 ∇ 20 20 ∇ 21 21 ∇ 22 22 ∇ 23 23 ∇ 24 24 ∇ 25 25 ∇ 26 26 ∇ 27 27 ∇ 28 28 ∇ 29 29 ∇ 30 30 ∇ 31 31 c_caldays; text; SAS:$CALDAY ARC arc_default
Start Date - Month* i_cmstmo; text3; SAS:CMSTMO	∇ UNK Unknown ∇ JAN Jan ∇ FEB Feb ∇ MAR Mar ∇ APR Apr ∇ MAY May ∇ JUN Jun ∇ JUL Jul ∇ AUG Aug ∇ SEP Sep ∇ OCT Oct ∇ NOV Nov ∇ DEC Dec c_calmonu; text; SAS:$CALMONU ARC arc_default
Start Date - Year* i_cmstyr; integer4; SAS:CMSTYR	____ ARC arc_default
Ongoing?* i_cmongo; text7; SAS:CMONGO<br><br>If subject is deceased and medication was ongoing at the time of death, enter Ongoing = 'Yes'.	∇ Y Yes ∇ N No ∇ UNK Unknown c_nyunk; text; SAS:$NYUNK; display.style radio ARC arc_default
Stop Date - Day* i_cmendy; text2; SAS:CMENDY<br>ENABLE i_cmendy, i_cmenmo, i_cmenyr if i_cmongo = N	∇ UN Unknown ∇ 01 01 ∇ 02 02 ∇ 03 03 ∇ 04 04 ∇ 05 05 ∇ 06 06 ∇ 07 07 ∇ 08 08 ∇ 09 09 ∇ 10 10 ∇ 11 11 ∇ 12 12 ∇ 13 13 ∇ 14 14 ∇ 15 15 ∇ 16 16 ∇ 17 17 ∇ 18 18 ∇ 19 19 ∇ 20 20 ∇ 21 21 ∇ 22 22 ∇ 23 23 ∇ 24 24 ∇ 25 25 ∇ 26 26 ∇ 27 27 ∇ 28 28 ∇ 29 29 ∇ 30 30 ∇ 31 31 c_caldays; text; SAS:$CALDAY ARC arc_default
Stop Date - Month* i_cmenmo; text3; SAS:CMENMO<br>ENABLE i_cmendy, i_cmenmo, i_cmenyr if i_cmongo = N	∇ UNK Unknown ∇ JAN Jan ∇ FEB Feb ∇ MAR Mar ∇ APR Apr ∇ MAY May ∇ JUN Jun ∇ JUL Jul ∇ AUG Aug ∇ SEP Sep ∇ OCT Oct ∇ NOV Nov ∇ DEC Dec c_calmonu; text; SAS:$CALMONU ARC arc_default
Stop Date - Year* i_cmenyr; integer4; SAS:CMENYR<br>ENABLE i_cmendy, i_cmenmo, i_cmenyr if i_cmongo = N	____ ARC arc_default
Dose* i_cmdstxt; text20; SAS:CMDSTXT	____________________ ARC arc_default
Dose Units* i_cmdosu; text10; SAS:CMDOSU	∇ mg mg ∇ ug ug ∇ mL mL ∇ g g ∇ IU IU ∇ TABLET tablet ∇ CAPSULE capsule ∇ mg/kg mg/kg ∇ OTHER Other ∇ UNKNOWN Unknown c_cmdosu; text; SAS:$CMDOSU ARC arc_default
Other Dose Units* i_cmdosuo; text50; SAS:CMDOSUO<br>ENABLE i_cmdosuo if i_cmdos = OTHER	______________________________ ARC arc_default

Concomitant Medication* *g_cm; SAS:CM*

Frequency* *i_cmfreq; text10; SAS:CMFREQ*	∇ *QD* Daily ∇ *BID* Twice Daily ∇ *TID* Three Times Daily ∇ *QID* Four Times Daily ∇ *QOD* Every Other Day ∇ *QM* Monthly ∇ *PRN* As Necessary ∇ *UNKNOWN* Unknown ∇ *OTHER* Other *c_dosfrq; text; SAS:$DOSFRQ* *ARC arc_default*
Other Frequency* *i_cmfreqo; text50; SAS:CMFREQO* *ENABLE i_cmfreqo if i_cmfreq = OTHER*	_ *ARC arc_default*
Route* *i_cmroute; text25; SAS:CMROUTE*	∇ *ORAL* Oral ∇ *INTRAVENOUS* Intravenous ∇ *SUBCUTANEOUS* Subcutaneous ∇ *INTRAMUSCULAR* Intramuscular ∇ *OTHER* Other ∇ *UNKNOWN* Unknown *c_cmroute; text; SAS:$CMROUTE* *ARC arc_default*
Other Route* *i_cmrouteo; text50; SAS:CMROUTEO* *ENABLE i_cmrouteo if i_cmroute = OTHER*	_ *ARC arc_default*

Intra-operative and Post-operative Transfusions * *e_TRANS; Scheduled*

CALCULATEEVENT: create e_TRANS event when i_subjid is assigned by copying i_subjid into i_subjidcopy on the f_subjcopy form within the e_TRANS event.

Subject ID* *f_subjcopy*

- [Form already referred to in a previous event!]

Transfusions* *f_trans*

ENABLE f_trans form if i_transiyn = Y or i_transpyn = Y

Intra-operative and Post-operative Transfusions* *g_transyn; SAS:TRANSYN*

Did the subject receive any intra-operative transfusions?* *i_transiyn; text1; SAS:TRANSIYN*	∇ *Y* Yes ∇ *N* No *c_ny; text; SAS:$NY; display.style radio* *ARC arc_default*
Did the subject receive any post-operative transfusions up to 72 hours post surgery?* *i_transpyn; text3; SAS:TRANSPYN*	∇ *Y* Yes ∇ *N* No ∇ *UNK* Unknown *c_nyunk; text; SAS:$NYUNK; display.style radio* *ARC arc_default*

If "Yes", click 'Add ItemGroup' to record each intra-operative and post-operative transfusion (up to 72 hours post surgery) below: [# _ _]* *g_trans; SAS:TRANSP; display.style horizontal*

ENABLE g_trans item group if (i_transiyn = Y or i_transpyn = Y)

Date of Transfusion* *i_trandat; date10; SAS:TRANDAT*	_ _ /_ _ /_ _ _ _ mm/dd/yyyy *ARC arc_default*
Time of Transfusion* *i_trantim; time5; SAS:TRANTIM*	_ _ :_ _ hh:mm *ARC arc_default*
Blood Product Type* *i_trantyp; text50; SAS:TRANTYP*	∇ *AUTOLOGOUS BLOOD TRANSFUSION/CELL SALVAGE* Autologous blood transfusion/cell salvage ∇ *ALLOGENEIC PLATELET CONCENTRATES* Allogeneic Platelet Concentrates ∇ *ALLOGENEIC RBCS* Allogeneic RBCs ∇ *ALLOGENEIC FRESH FROZEN PLASMA (FFP)* Allogeneic Fresh Frozen Plasma (FFP) ∇ *OTHER* Other ∇ *UNKNOWN* Unknown *c_trantyp; text; SAS:$TRANTYP* *ARC arc_default*
Volume of Blood Product Transfused* *i_tranvol; integer4; SAS:TRANVOL*	_ _ _ _ mL *mu_ml* *ARC arc_default*

*Post-operative Surgical Revisions/Re-operations** *e_PRREVP; Scheduled*

CALCULATEEVENT: create e_PRREVP event when i_subjid is assigned by copying i_subjid into i_subjidcopy on the f_subjcopy form within the e_PRREVP event.

Subject ID* *f_subjcopy*

- [Form already referred to in a previous event!]

Post-operative Surgical Revisions/Re-operations* *f_prrevp*

Post-operative Surgical Revisions/Re-operations* *g_prrevpyn; SAS:PRREVPYN*

Were any post-operative surgical revisions/re-operations performed due to bleeding, air or other body fluid leakage?* *i_prrevpyn; text3; SAS:PRREVPYN*	∇ Y Yes ∇ N No ∇ UNK Unknown *c_nyunk; text; SAS:$NYUNK; display.style radio* ARC arc_default

If "Yes", please click 'Add ItemGroup' to record each post-operative surgical procedure: [# _ _]* *g_prrevp; SAS:PRREVP; display.style horizontal*

ENABLE g_prrevp item group if i_prrevpyn = Y

Procedure Description* *i_prrevterm; text200; SAS:PRREVTRM*	_ (×9 lines) ARC arc_default
Indication* *i_prrevindc; text200; SAS:PRREVIND*	_ (×9 lines) ARC arc_default
Start Date* *i_prrevsdat; date10; SAS:PRREVSDT*	_ _ / _ _ / _ _ _ _ mm/dd/yyyy ARC arc_default
Start Time* *i_prrevstim; time5; SAS:PRREVSTM*	_ _ : _ _ hh:mm ARC arc_default
End Date* *i_prrevedat; date10; SAS:PRREVEDT*	_ _ / _ _ / _ _ _ _ mm/dd/yyyy ARC arc_default
End Time* *i_prrevetim; time5; SAS:PRREVETM*	_ _ : _ _ hh:mm ARC arc_default

*Hepatobiliary Surgery: {0}** *e_LIVER; Scheduled; EP(0): FormData[@OID="f_cohortid"]/ItemGroupData[@OID="g_cohortid"]/ItemData[@OID="i_cohortid"]*

CALCULATEEVENT: Create e_LIVER event when Cohort ID for Hepatobiliary Surgery is assigned (i_colivid), by copying the i_colivid into i_cohortid within the e_LIVER event (more details to be provided in dynamic check plan)

HEMOPATCH Indication Cohort ID* *f_cohortid*

HEMOPATCH Indication Cohort ID* *g_cohortid; SAS:COHORTID*

Indication Cohort ID* *i_cohortid; text10; SAS:COHORTID* *CALCULATEONCE on each surgery-specific form based on cohorts selected on f_surgery form. (details to be provided later in dynamic check plan)*	_ _ _ _ _ _ _ _ _ _ *ARC arc_default*

Intra-operative Assessment: Hepatobiliary Surgery* *f_liver*

HEMOPATCH Application Details and Sealing Assessment* *g_hemo; SAS:HEMO*

Target Application Site (TAS) Location and Tissue Type* *i_tasloc; text200; SAS:TASLOC*	_ (9 lines) *ARC arc_default*
Number of HEMOPATCH units applied* *i_patchnum; integer2; SAS:PATCHNUM*	_ _ Unit(s) *mu_patch ARC arc_default*
If multiple HEMOPATCH units were applied, please provide the main reason:* *i_patchreas; text200; SAS:PATCHREA* *ENABLE i_patchreas if i_patchnum > 1*	_ (9 lines) *ARC arc_default*
Was any slipping of HEMOPATCH from the TAS observed during surgery?* *i_leakyn; text1; SAS:LEAKYN*	∇ Y Yes ∇ N No *c_ny; text; SAS:$NY; display.style radio* *ARC arc_default*
Time to Hemostasis* *i_hemodur; integer3; SAS:HEMODUR*	_ _ _ Seconds *mu_sec ARC arc_default*
Were there any re-bleeds from the time hemostasis was achieved until the end of surgery?** *i_hemorbld; text1; SAS:HEMORBLD*	∇ Y Yes ∇ N No *c_ny; text; SAS:$NY; display.style radio* *ARC arc_default*

HEMOPATCH Application Details and Sealing Assessment* *g_hemo; SAS:HEMO*

Were any intra-operative surgical revisions required due to bleeding, air, or other body fluid leakage after primary hemostasis was achieved?* *i_prreviyn; text1; SAS:PRREVIYN*	∇ Y Yes ∇ N No *c_ny; text; SAS:$NY; display.style radio*<br>*ARC arc_default*
If "Yes", please describe revisions:* *i_prrevi; text200; SAS:PRREVI*<br>*ENABLE i_prrevi if i_prreviyn = Y*	_ _<br>_ _<br>_ _<br>_ _<br>_ _<br>_ _<br>_ _<br>_ _<br>_ _ _ _ _ _ _ _ _ _ _ _ _ _ _ _ *ARC arc_default*
Were any other hemostatic agents or sealants used at the TAS?* *i_hemoagyn; text1; SAS:HEMOAGYN*	∇ Y Yes ∇ N No *c_ny; text; SAS:$NY; display.style radio*<br>*ARC arc_default*
	*Any re-bleeds should be recorded as an Adverse Event.

If any other hemostatic agents or sealants were used at the TAS, please use 'Add Item Group' to record each below: [# _ _]* ***g_hemoag; SAS:HEMOAG; display.style horizontal***
ENABLE g_hemoag item group if i_hemoagyn = Y

Name of Hemostatic Agent / Sealant* *i_hemoagnt; text200; SAS:HEMOAGNT*	_ _<br>_ _<br>_ _<br>_ _<br>_ _<br>_ _<br>_ _<br>_ _<br>_ _ _ _ _ _ _ _ _ _ _ _ _ _ _ _ *ARC arc_default*

Bicarbonate Use* *f_bicarb*

Bicarbonate Use* *g_bicarb; SAS:BICARB*

Did you decide to use bicarbonate with the application of HEMOPATCH?* *i_bcyn; text1; SAS:BCYN*<br>*ENABLE i_bcreas, i_bcapp, i_bcconc, and i_bcadhr if i_bcyn = Y*	∇ Y Yes ∇ N No *c_ny; text; SAS:$NY; display.style radio*<br>*ARC arc_default*
Why do you use bicarbonate in combination with the application of HEMOPATCH?* *i_bcreas; text50; SAS:BCREAS*	∇ I ALWAYS USE IT I always use it ∇ TISSUE SURFACE WAS TOO DRY Tissue surface was too dry ∇ OTHER Other *c_bcreas; text; SAS:$BCREAS*<br>*ARC arc_default*

Bicarbonate Use* g_bicarb; SAS:BICARB

If "Other", please specify other reason for using bicarbonate with the application of HEMOPATCH:* i_bcreaso; text200; SAS:BCREASO<br>ENABLE i_bcreaso if i_bcreas = OTHER	_ _<br>_ _<br>_ _<br>_ _<br>_ _<br>_ _<br>_ _<br>_ _<br>_ _ _ _ _ _ _ _ _ _ _ _ _ _ _ _ ARC arc_default
How was bicarbonate applied?* i_bcapp; text50; SAS:BCAPP	∇ APPLIED DIRECTLY TO TISSUE SURFACE Applied directly to tissue surface ∇ APPLIED TO INACTIVE SURFACE OF PATCH BY WET GAUZE Applied to inactive (blue-dot) surface of HEMOPATCH using gauze after application of HEMOPATCH to the wound ∇ APPLIED TO INACTIVE SURFACE BY DRIP SOLUTION Applied to inactive (blue-dot) surface of HEMOPATCH using dripping solution after application of HEMOPATCH to the wound ∇ APPLIED DIRECTLY TO ACTIVE SIDE OF PATCH Applied directly to active side (no blue dots) of HEMOPATCH immediately before applying HEMOPATCH to the tissue surface c_bcapp; text; SAS:$BCAPP<br>ARC arc_default
What concentration of bicarbonate did you use?* i_bcconc; text6; SAS:BCCONC	∇ 4.2% 4.2% ∇ 8.4% 8.4% ∇ OTHER Other c_bcconc; text; SAS:$BCCONC<br>ARC arc_default
If "Other", please specify concentration:* i_bcconco; float3,1; SAS:BCCONCO<br>ENABLE i_bcconco if i_bcconc = OTHER	_ _ , _ % m_percent ARC arc_default
Did the use of bicarbonate result in adequate adherence?* i_bcadhr; text3; SAS:BCADHR	∇ Y Yes ∇ N No ∇ UNK Unknown c_nyunk; text; SAS:$NYUNK; display.style radio<br>ARC arc_default

Post-operative Follow-up Week 1 - {0}* f_fu1; EP(0): ItemGroupData[@OID="g_fu"]/ItemData[@OID="i_fudat"]

Post-operative Weekly Follow-up Assessment* g_fu; SAS:FU

Date of Assessment* i_fudat; date10; SAS:FUDAT	_ _ /_ _ /_ _ _ _ mm/dd/yyyy ARC arc_default
Did the subject experience any allergic reactions to HEMOPATCH at the TAS during this week of follow-up?* i_alrgyyn; text3; SAS:ALRGYYN	∇ Y Yes ∇ N No ∇ UNK Unknown c_nyunk; text; SAS:$NYUNK; display.style radio<br>ARC arc_default
Did the subject experience any re-bleeding at the TAS during this week of follow-up?* i_rebldyn; text3; SAS:REBLD	∇ Y Yes ∇ N No ∇ UNK Unknown c_nyunk; text; SAS:$NYUNK; display.style radio<br>ARC arc_default
Did the subject experience a hematoma at the TAS during this week of follow-up?* i_hemayn; text3; SAS:HEMAYN	∇ Y Yes ∇ N No ∇ UNK Unknown c_nyunk; text; SAS:$NYUNK; display.style radio<br>ARC arc_default
Were there any signs of an abnormal infection at the TAS during this week of follow-up?* i_infectyn; text3; SAS:INFECTYN	∇ Y Yes ∇ N No ∇ UNK Unknown c_nyunk; text; SAS:$NYUNK; display.style radio<br>ARC arc_default
	If "Yes" to any of the above, please record an Adverse Event.

Post-operative Follow-up Week 2 - {0}* *f_fu2; EP(0): ItemGroupData[@OID="g_fu"]/ItemData[@OID="i_fudat"]*

Post-operative Weekly Follow-up Assessment* *g_fu; SAS:FU*

- [Itemgroup already referred to in a previous form!]

Post-operative Follow-up Week 3 - {0}* *f_fu3; EP(0): ItemGroupData[@OID="g_fu"]/ItemData[@OID="i_fudat"]*

Post-operative Weekly Follow-up Assessment* *g_fu; SAS:FU*

- [Itemgroup already referred to in a previous form!]

Post-operative Follow-up Week 4 - {0}* *f_fu4; EP(0): ItemGroupData[@OID="g_fu"]/ItemData[@OID="i_fudat"]*

Post-operative Weekly Follow-up Assessment* *g_fu; SAS:FU*

- [Itemgroup already referred to in a previous form!]

Post-operative Follow-up: Hepatobiliary Surgery* *f_fuliv*

Please complete the following overall assessment at the end of the follow-up period.

Hepatobiliary Surgery Follow-up Assessments* *g_fuliv; SAS:FULIV*

During the 4-week follow-up period, did the subject experience any post-operative pancreatic fistulas?* *i_fulivq1; text3; SAS:FULIVQ1*	∇ Y Yes ∇ N No ∇ UNK Unknown *c_nyunk; text; SAS:$NYUNK; display.style radio* *ARC arc_default*
Was there any post-operative bile leakage?* *i_fulivq2; text3; SAS:FULIVQ2*	∇ Y Yes ∇ N No ∇ UNK Unknown *c_nyunk; text; SAS:$NYUNK; display.style radio* *ARC arc_default*
If "Yes", please provide the duration of post-operative Bile Leakage:* *i_fulivq3; integer2; SAS:FULIVQ3* *ENABLE i_fulivq3 if i_fulivq2 = Y*	_ _ Day(s) *mu_days ARC arc_default*

*General Surgery: {0}** *e_GENERAL; Scheduled; EP(0): FormData[@OID="f_cohortid"]/ItemGroupData[@OID="g_cohortid"]/ItemData[@OID="i_cohortid"]*

CALCULATEEVENT: Create e_GENERAL event when Cohort ID for General Surgery is assigned (i_cogenid), by copying the i_cogenid into i_cohortid within the e_GENERAL event (more details to be provided in dynamic check plan)

HEMOPATCH Indication Cohort ID* *f_cohortid*

- [Form already referred to in a previous event!]

Intra-operative Assessment: General Surgery* *f_general*

HEMOPATCH Application Details and Sealing Assessment* *g_hemo; SAS:HEMO*

- [Itemgroup already referred to in a previous form!]

If any other hemostatic agents or sealants were used at the TAS, please use 'Add Item Group' to record each below: [# _ _]* *g_hemoag; SAS:HEMOAG; display.style horizontal*

ENABLE g_hemoag item group if i_hemoagyn = Y

- [Itemgroup already referred to in a previous form!]

Bicarbonate Use* *f_bicarb*

- [Form already referred to in a previous event!]

Post-operative Follow-up Week 1 - {0}* *f_fu1; EP(0): ItemGroupData[@OID="g_fu"]/ItemData[@OID="i_fudat"]*

- [Form already referred to in a previous event!]

Post-operative Follow-up Week 2 - {0}* *f_fu2; EP(0): ItemGroupData[@OID="g_fu"]/ItemData[@OID="i_fudat"]*

- [Form already referred to in a previous event!]

Post-operative Follow-up Week 3 - {0}* *f_fu3; EP(0): ItemGroupData[@OID="g_fu"]/ItemData[@OID="i_fudat"]*

- [Form already referred to in a previous event!]

Post-operative Follow-up Week 4 - {0}* *f_fu4; EP(0): ItemGroupData[@OID="g_fu"]/ItemData[@OID="i_fudat"]*

- [Form already referred to in a previous event!]

Post-operative Follow-up: General Surgery* *f_fugen*

Please complete the following overall assessment at the end of the follow-up period.

General Surgery Follow-up Assessments* *g_fugen; SAS:FUGEN*

During the 4-week follow-up period, did the subject experience any GI anastomosis leakage/fistula?* *i_fugenq1; text3; SAS:FUGENQ1*	∇ *Y* Yes ∇ *N* No ∇ *N/A* Not Applicable ∇ *UNK* Unknown *c_nyunkna; text; SAS:$NYUNKNA; display.style radio* *ARC arc_default*

*Thoracic/Lung Surgery: {0}** *e_LUNG; Scheduled; EP(0): FormData[@OID="f_cohortid"]/ItemGroupData[@OID="g_cohortid"]/ItemData[@OID="i_cohortid"]*

CALCULATEEVENT: Create e_LUNG event when Cohort ID for Lung/Thoracic Surgery is assigned (i_colungid), by copying the i_colungid into i_cohortid within the e_LUNG event (more details to be provided in dynamic check plan)

HEMOPATCH Indication Cohort ID* *f_cohortid*

- [Form already referred to in a previous event!]

Intra-operative Assessment: Thoracic/Lung Surgery* *f_lung*

HEMOPATCH Application Details and Sealing Assessment* *g_hemo; SAS:HEMO*

- [Itemgroup already referred to in a previous form!]

If any other hemostatic agents or sealants were used at the TAS, please use 'Add Item Group' to record each below: [# _ _]* *g_hemoag; SAS:HEMOAG; display.style horizontal*

ENABLE g_hemoag item group if i_hemoagyn = Y

- [Itemgroup already referred to in a previous form!]

Thoracic/Lung Surgery Intra-operative Assessment* *g_lung; SAS:LUNG*

If HEMOPATCH was applied to the lung, was an air-tight seal achieved during surgery?* *i_lungq1; text3; SAS:LUNGQ1*	∇ *Y* Yes ∇ *N* No ∇ *N/A* Not Applicable ∇ *UNK* Unknown *c_nyunkna; text; SAS:$NYUNKNA; display.style radio* *ARC arc_default*

Bicarbonate Use* *f_bicarb*

- [Form already referred to in a previous event!]

Post-operative Follow-up Week 1 - {0}* *f_fu1; EP(0): ItemGroupData[@OID="g_fu"]/ItemData[@OID="i_fudat"]*

- [Form already referred to in a previous event!]

Post-operative Follow-up Week 2 - {0}* *f_fu2; EP(0): ItemGroupData[@OID="g_fu"]/ItemData[@OID="i_fudat"]*

- [Form already referred to in a previous event!]

Post-operative Follow-up Week 3 - {0}* *f_fu3; EP(0): ItemGroupData[@OID="g_fu"]/ItemData[@OID="i_fudat"]*

- [Form already referred to in a previous event!]

Post-operative Follow-up Week 4 - {0}* *f_fu4; EP(0): ItemGroupData[@OID="g_fu"]/ItemData[@OID="i_fudat"]*

- [Form already referred to in a previous event!]

Post-operative Follow-up: Thoracic/Lung Surgery* *f_fulung*

Please complete the following overall assessment at the end of the follow-up period.

Thoracic/Lung Surgery Follow-up Assessments* *g_fulung; SAS:FULUNG*

Duration of chest tube drainage* *i_fulungq1; integer3; SAS:FULUNGQ1*	_ _ _ Day(s) *mu_days ARC arc_default*
Did the chest tube need to be reinserted for pneumothorax?* *i_fulungq2; text3; SAS:FULUNGQ2*	∇ Y Yes ∇ N No *c_ny; text; SAS:$NY; display.style radio ARC arc_default*

*Cardiovascular Surgery: {0}** *e_CARDIO; Scheduled; EP(0): FormData[@OID="f_cohortid"]/ItemGroupData[@OID="g_cohortid"]/ItemData[@OID="i_cohortid"]*

CALCULATEEVENT: Create e_CARDIO event when Cohort ID for Cardiovascular Surgery is assigned (i_cocardid), by copying the i_cocardid into i_cohortid within the e_CARDIO event (more details to be provided in dynamic check plan)

HEMOPATCH Indication Cohort ID* *f_cohortid*

- [Form already referred to in a previous event!]

Intra-operative Assessment: Cardiovascular Surgery* *f_cardio*

HEMOPATCH Application Details and Sealing Assessment* *g_hemo; SAS:HEMO*

- [Itemgroup already referred to in a previous form!]

If any other hemostatic agents or sealants were used at the TAS, please use 'Add Item Group' to record each below: [# _ _]* *g_hemoag; SAS:HEMOAG; display.style horizontal*

ENABLE g_hemoag item group if i_hemoagyn = Y

- [Itemgroup already referred to in a previous form!]

Bicarbonate Use* *f_bicarb*

- [Form already referred to in a previous event!]

Post-operative Follow-up Week 1 - {0}* *f_fu1; EP(0): ItemGroupData[@OID="g_fu"]/ItemData[@OID="i_fudat"]*

- [Form already referred to in a previous event!]

Post-operative Follow-up Week 2 - {0}* *f_fu2; EP(0): ItemGroupData[@OID="g_fu"]/ItemData[@OID="i_fudat"]*

- [Form already referred to in a previous event!]

Post-operative Follow-up Week 3 - {0}* *f_fu3; EP(0): ItemGroupData[@OID="g_fu"]/ItemData[@OID="i_fudat"]*

- [Form already referred to in a previous event!]

Post-operative Follow-up Week 4 - {0}* *f_fu4; EP(0): ItemGroupData[@OID="g_fu"]/ItemData[@OID="i_fudat"]*

- [Form already referred to in a previous event!]

Post-operative Assessments: Cardiovascular Surgery* *f_fucardio*

Please complete the following overall assessment at the end of the follow-up period.

Cardiovascular Surgery Follow-up Assessments* *g_fucardio; SAS:FUCARDIO*

Duration of chest tube drainage* *i_fucardioq1; integer2; SAS:FUCARQ1*	_ _ Day(s) *mu_days ARC arc_default*
Did the chest tube need to be reinserted for pneumothorax?* *i_fucardioq2; text3; SAS:FUCARQ2*	∇ *Y* Yes ∇ *N* No ∇ *UNK* Unknown *c_nyunk; text; SAS:$NYUNK; display.style radio* *ARC arc_default*

*Neurological/Spinal Surgery: {0}** *e_NEURO; Scheduled; EP(0): FormData[@OID="f_cohortid"]/ItemGroupData[@OID="g_cohortid"]/ItemData[@OID="i_cohortid"]*

CALCULATEEVENT: Create e_NEURO event when Cohort ID for Neurological/Spinal Surgery is assigned (i_coneurid), by copying the i_coneurid into i_cohortid within the e_NEURO event (more details to be provided in dynamic check plan)

HEMOPATCH Indication Cohort ID* *f_cohortid*

- [Form already referred to in a previous event!]

Intra-operative Assessment: Neurological/Spinal Surgery* *f_neuro*

HEMOPATCH Application Details and Sealing Assessment* *g_hemo; SAS:HEMO*

- [Itemgroup already referred to in a previous form!]

If any other hemostatic agents or sealants were used at the TAS, please use 'Add Item Group' to record each below: [# _ _]* *g_hemoag; SAS:HEMOAG; display.style horizontal*

ENABLE g_hemoag item group if i_hemoagyn = Y

- [Itemgroup already referred to in a previous form!]

Neurological/Spinal Surgery Intra-operative Assessment* *g_neuro; SAS:NEURO*

If HEMOPATCH was applied to the dura, was a water-tight closure achieved?* *i_neurq1; text3; SAS:NEURQ1*	∇ *Y* Yes ∇ *N* No ∇ *N/A* Not Applicable ∇ *UNK* Unknown *c_nyunkna; text; SAS:$NYUNKNA; display.style radio* *ARC arc_default*

Bicarbonate Use* *f_bicarb*

- [Form already referred to in a previous event!]

Post-operative Follow-up Week 1 - {0}* *f_fu1; EP(0): ItemGroupData[@OID="g_fu"]/ItemData[@OID="i_fudat"]*

- [Form already referred to in a previous event!]

Post-operative Follow-up Week 2 - {0}* *f_fu2; EP(0): ItemGroupData[@OID="g_fu"]/ItemData[@OID="i_fudat"]*

- [Form already referred to in a previous event!]

Post-operative Follow-up Week 3 - {0}* *f_fu3; EP(0): ItemGroupData[@OID="g_fu"]/ItemData[@OID="i_fudat"]*

- [Form already referred to in a previous event!]

Post-operative Follow-up Week 4 - {0}* *f_fu4; EP(0): ItemGroupData[@OID="g_fu"]/ItemData[@OID="i_fudat"]*

- [Form already referred to in a previous event!]

Post-operative Follow-up: Neurological Surgery* *f_funeuro*

Please complete the following overall assessment at the end of the follow-up period.

Neurological Surgery Follow-up Assessments* *g_funeuro; SAS:FUNEURO*

During the 4-week follow-up period, was there any post-operative CSF leakage?* *i_funeurq1; text3; SAS:FUNEURQ1*	∇ *Y* Yes ∇ *N* No ∇ *UNK* Unknown *c_nyunk; text; SAS:$NYUNK; display.style radio* *ARC arc_default*

*Urological Surgery: {0}**e_UROLOGIC; Scheduled; EP(0): FormData[@OID="f_cohortid"]/ItemGroupData[@OID="g_cohortid"]/ItemData[@OID="i_cohortid"]*

CALCULATEEVENT: Create e_UROLOGIC event when Cohort ID for Urological Surgery is assigned (i_couroid), by copying the i_couroid into i_cohortid within the e_UROLOGIC event (more details to be provided in dynamic check plan)

HEMOPATCH Indication Cohort ID* *f_cohortid*

- [Form already referred to in a previous event!]

Intra-operative Assessment: Urological Surgery* *f_urologic*

HEMOPATCH Application Details and Sealing Assessment* *g_hemo; SAS:HEMO*

- [Itemgroup already referred to in a previous form!]

If any other hemostatic agents or sealants were used at the TAS, please use 'Add Item Group' to record each below: [# _ _]* *g_hemoag; SAS:HEMOAG; display.style horizontal*

ENABLE g_hemoag item group if i_hemoagyn = Y

- [Itemgroup already referred to in a previous form!]

Bicarbonate Use* *f_bicarb*

- [Form already referred to in a previous event!]

Post-operative Follow-up Week 1 - {0}* *f_fu1; EP(0): ItemGroupData[@OID="g_fu"]/ItemData[@OID="i_fudat"]*

- [Form already referred to in a previous event!]

Post-operative Follow-up Week 2 - {0}* *f_fu2; EP(0): ItemGroupData[@OID="g_fu"]/ItemData[@OID="i_fudat"]*

- [Form already referred to in a previous event!]

Post-operative Follow-up Week 3 - {0}* *f_fu3; EP(0): ItemGroupData[@OID="g_fu"]/ItemData[@OID="i_fudat"]*

- [Form already referred to in a previous event!]

Post-operative Follow-up Week 4 - {0}* *f_fu4; EP(0): ItemGroupData[@OID="g_fu"]/ItemData[@OID="i_fudat"]*

- [Form already referred to in a previous event!]

Post-operative Follow-up: Urological Surgery* *f_fuuro*

Please complete the following overall assessment at the end of the follow-up period.

Urological Surgery Follow-up Assessments* *g_fuuro; SAS:FUURO*

During the 4-week follow-up period, was there any post-operative urinary fistula formation?* *i_fuuroq1; text3; SAS:FUUROQ1*	∇ *Y* Yes ∇ *N* No ∇ *UNK* Unknown *c_nyunk; text; SAS:$NYUNK; display.style radio* *ARC arc_default*

End of Study * *e_EOS; Scheduled*

CALCULATEEVENT: create e_EOS event when i_subjid is assigned by copying i_subjid into i_subjidcopy on the f_subjcopy form within the e_EOS event.

Subject ID* *f_subjcopy*

- [Form already referred to in a previous event!]

Disposition - End of Study* *f_ds*

Disposition - End of Study* *g_ds; SAS:DS*

Subject Status:* *i_dsstatus; text12; SAS:DSSTATUS*	∇ *COMPLETED* Completed ∇ *DISCONTINUED* Discontinued *c_dsstat; text; SAS:$DSSTAT* *ARC arc_default*
Date of Completion/Discontinuation* *i_dsstdat; date10; SAS:DSSTDAT*	_ _ / _ _ / _ _ _ _ mm/dd/yyyy *ARC arc_default*
If "Discontinued", please provide the primary reason for withdrawal:* *i_dsdecod; text30; SAS:DSDECOD* *ENABLE i_dsdecod if i_dsstatus = DISCONTINUED*	∇ *ADVERSE EVENT* Adverse Event ∇ *LOST TO FOLLOW-UP* Lost to Follow-up ∇ *VOLUNTARY WITHDRAWAL* Voluntary Withdrawal ∇ *INVESTIGATOR DISCRETION* Investigator Discretion ∇ *TERMINATION OF STUDY* Termination of Study ∇ *OTHER* Other *c_ncomplt; text; SAS:$NCOMPLT* *ARC arc_default*
If "Other", please specify:* *i_dsterm; text200; SAS:DSTERM* *ENABLE i_dsterm if i_dsdecod = OTHER*	_ *ARC arc_default*

Administrative Protocol: Medical Center Characteristics

p_admin1

*Medical Center Characteristics** *e_admin1_MEDCENTER; Scheduled*

Medical Center Characteristics* *f_admin1_medcenter*

Medical Center Characteristics* *g_admin1_medcenter; SAS:MEDCENT*

Date of Assessment* *i_admin1_mcdat; date10; SAS:MCDAT*	_ _ /_ _ /_ _ _ _ mm/dd/yyyy *ARC arc_default*
Number of Beds* *i_admin1_mcbednum; integer5; SAS:MCBEDNUM*	_ _ _ _ _ Beds *mu_beds ARC arc_default*
Type of Institution* *i_admin1_mctype; text50; SAS:MCTYPE*	∇ *PUBLIC* Public ∇ *PRIVATE* Private *c_mctype; text; SAS:$MCTYPE ARC arc_default*
Number of Hepatobiliary Surgeries performed annually:* *i_admin1_livnum; integer5; SAS:LIVNUM*	_ _ _ _ _ Surgeries *mu_surgeries ARC arc_default*
Number of General Surgeries performed annually:* *i_admin1_gennum; integer5; SAS:GENNUM*	_ _ _ _ _ Surgeries *mu_surgeries ARC arc_default*
Number of Thoracic/Lung Surgeries performed annually:* *i_admin1_lungnum; integer5; SAS:LUNGNUM*	_ _ _ _ _ Surgeries *mu_surgeries ARC arc_default*
Number of Cardiovascular Surgeries performed annually:* *i_admin1_cardnum; integer5; SAS:CARDNUM*	_ _ _ _ _ Surgeries *mu_surgeries ARC arc_default*
Number of Neurological/Spinal Surgeries performed annually:* *i_admin1_neurnum; integer5; SAS:NEURNUM*	_ _ _ _ _ Surgeries *mu_surgeries ARC arc_default*
Number of Urological Surgeries performed annually:* *i_admin1_uronum; integer5; SAS:URONUM*	_ _ _ _ _ Surgeries *mu_surgeries ARC arc_default*

Administrative Protocol: HEMOPATCH Questionnaire

p_admin2_surgeon

*HEMOPATCH Intraoperative Use Evaluation Questionnaire** *e_admin2_SURGEON; Scheduled*

HEMOPATCH Questionnaire Surgeon 1: {0} {1} *f_admin2_1surgeon; EP(0): ItemGroupData[@OID="g_admin2_surgid"]/ItemData[@OID="i_admin2_nam1"]; EP(1): ItemGroupData[@OID="g_admin2_surgid"]/ItemData[@OID="i_admin2_nam2"]*

HEMOPATCH Intraoperative Use Evaluation Questionnaire* *g_admin2_surgid; SAS:A2SRGID*

First Name:* *i_admin2_nam1; text20; SAS:A2NAM1*	_ ARC<br>arc_default
Last Name:* *i_admin2_nam2; text20; SAS:A2NAM2*	_ ARC<br>arc_default
Title:* *i_admin2_nam3; text20; SAS:A2NAM3*	_ ARC<br>arc_default
Hospital and Department* *i_admin2_Hosp; text200; SAS:A2HOSP*	_ _<br>_ _<br>_ _<br>_ _<br>_ _<br>_ _<br>_ _<br>_ _<br>_ _ _ _ _ _ _ _ _ _ _ _ _ _ _ _ ARC arc_default
How many times have you applied HEMOPATCH Sealing Haemostat before?* *i_admin2_hemo; text9; SAS:A2HEMO*	∇ 0 0 ∇ 1 1 ∇ 2 2 ∇ 3 3 ∇ 4 or more 4 or more *c_app; text; SAS:$app*<br>ARC arc_default
Have you been trained by Baxter on the product?* *i_admin2_train; text1; SAS:A2TRAIN*	∇ Y Yes ∇ N No *c_ny; text; SAS:$NY; display.style radio*<br>ARC arc_default

Could you please rate HEMOPATCH on the following features?* *g_admin2_q5; SAS:A2Q5*

Ease of preparation* *i_admin2_q5_1; text50; SAS:A2Q5_1*	∇ EXCELLENT Excellent ∇ GOOD Good ∇ MODERATE Moderate ∇ POOR Poor ∇ NOT APPLICABLE Not Applicable *c_feat; text; SAS:$FEAT; display.style radio*<br>ARC arc_default
Ease of handling* *i_admin2_q5_2; text50; SAS:A2Q5_2*	∇ EXCELLENT Excellent ∇ GOOD Good ∇ MODERATE Moderate ∇ POOR Poor ∇ NOT APPLICABLE Not Applicable *c_feat; text; SAS:$FEAT; display.style radio*<br>ARC arc_default
Tissue adherence* *i_admin2_q5_3; text50; SAS:A2Q5_3*	∇ EXCELLENT Excellent ∇ GOOD Good ∇ MODERATE Moderate ∇ POOR Poor ∇ NOT APPLICABLE Not Applicable *c_feat; text; SAS:$FEAT; display.style radio*<br>ARC arc_default

Could you please rate HEMOPATCH on the following features?* *g_admin2_q5; SAS:A2Q5*

Flexibility / Pliability* *i_admin2_q5_4; text50; SAS:A2Q5_4*	∇ *EXCELLENT* Excellent ∇ *GOOD* Good ∇ *MODERATE* Moderate ∇ *POOR* Poor ∇ *NOT APPLICABLE* Not Applicable *c_feat; text; SAS:$FEAT; display.style radio* *ARC arc_default*
Haemostatic efficacy* *i_admin2_q5_5; text50; SAS:A2Q5_5*	∇ *EXCELLENT* Excellent ∇ *GOOD* Good ∇ *MODERATE* Moderate ∇ *POOR* Poor ∇ *NOT APPLICABLE* Not Applicable *c_feat; text; SAS:$FEAT; display.style radio* *ARC arc_default*
Surgeon overall satisfaction* *i_admin2_q5_6; text50; SAS:A2Q5_6*	∇ *EXCELLENT* Excellent ∇ *GOOD* Good ∇ *MODERATE* Moderate ∇ *POOR* Poor ∇ *NOT APPLICABLE* Not Applicable *c_feat; text; SAS:$FEAT; display.style radio* *ARC arc_default*

What other haemostatic patches have you used before (check all that apply):* *g_admin2_q6; SAS:A2Q6*

Tachosil Patch *i_admin2_q6_1; text1; SAS:A2Q6_1*	☑ *Y* *c_checkyes; text; SAS:$CHKYES; display.style checkbox* *ARC arc_default*
Collagen Patch (e.g. TissuFleece, GentaFleece) *i_admin2_q6_2; text1; SAS:A2Q6_2*	☑ *Y* *c_checkyes; text; SAS:$CHKYES; display.style checkbox* *ARC arc_default*
Oxidized Cellulose Patch (e.g. Surgicel) *i_admin2_q6_3; text1; SAS:A2Q6_3*	☑ *Y* *c_checkyes; text; SAS:$CHKYES; display.style checkbox* *ARC arc_default*
Flowable Matrix (e.g. Floseal, Surgiflo) *i_admin2_q6_4; text1; SAS:A2Q6_4*	☑ *Y* *c_checkyes; text; SAS:$CHKYES; display.style checkbox* *ARC arc_default*
Sealant (e.g. Tisseel) *i_admin2_q6_5; text1; SAS:A2Q6_5*	☑ *Y* *c_checkyes; text; SAS:$CHKYES; display.style checkbox* *ARC arc_default*
Other *i_admin2_q6_6; text1; SAS:A2Q6_6*	☑ *Y* *c_checkyes; text; SAS:$CHKYES; display.style checkbox* *ARC arc_default*
Other, Specify* *i_admin2_q6_6A; text50; SAS:A2Q6_6A* *ENABLE i_admin2_q6_6A if i_admin2_q6_6 = Y*	_ *ARC arc_default*
None *i_admin2_q6_7; text1; SAS:A2Q6_7*	☑ *Y* *c_checkyes; text; SAS:$CHKYES; display.style checkbox* *ARC arc_default*

Compared to other haemostatic patches you have used, could you please rate HEMOPATCH on the following features? (Please tick only one box per line)* *g_admin2_q7; SAS:A2Q7*

Ease of preparation* *i_admin2_q7_1; text50; SAS:A2Q7_1*	∇ *MUCH BETTER* Much Better ∇ *BETTER* Better ∇ *EQUIVALENT* Equivalent ∇ *WORSE* Worse ∇ *NOT APPLICABLE* Not Applicable *c_feats; text; SAS:$FEATS; display.style radio* *ARC arc_default*
Ease of handling* *i_admin2_q7_2; text50; SAS:A2Q7_2*	∇ *MUCH BETTER* Much Better ∇ *BETTER* Better ∇ *EQUIVALENT* Equivalent ∇ *WORSE* Worse ∇ *NOT APPLICABLE* Not Applicable *c_feats; text; SAS:$FEATS; display.style radio* *ARC arc_default*
Flexibility / Pliability* *i_admin2_q7_3; text50; SAS:A2Q7_3*	∇ *MUCH BETTER* Much Better ∇ *BETTER* Better ∇ *EQUIVALENT* Equivalent ∇ *WORSE* Worse ∇ *NOT APPLICABLE* Not Applicable *c_feats; text; SAS:$FEATS; display.style radio* *ARC arc_default*
Tissue adherence* *i_admin2_q7_4; text50; SAS:A2Q7_4*	∇ *MUCH BETTER* Much Better ∇ *BETTER* Better ∇ *EQUIVALENT* Equivalent ∇ *WORSE* Worse ∇ *NOT APPLICABLE* Not Applicable *c_feats; text; SAS:$FEATS; display.style radio* *ARC arc_default*

Compared to other haemostatic patches you have used, could you please rate HEMOPATCH on the following features? (Please tick only one box per line)* *g_admin2_q7; SAS:A2Q7*

Haemostatic efficacy* *i_admin2_q7_5; text50; SAS:A2Q7_5*	∇ *MUCH BETTER* Much Better ∇ *BETTER* Better ∇ *EQUIVALENT* Equivalent ∇ *WORSE* Worse ∇ *NOT APPLICABLE* Not Applicable *c_feats; text; SAS:$FEATS; display.style radio* *ARC arc_default*
Surgeon overall satisfaction* *i_admin2_q7_6; text50; SAS:A2Q7_6*	∇ *MUCH BETTER* Much Better ∇ *BETTER* Better ∇ *EQUIVALENT* Equivalent ∇ *WORSE* Worse ∇ *NOT APPLICABLE* Not Applicable *c_feats; text; SAS:$FEATS; display.style radio* *ARC arc_default*

Would you like to use HEMOPATCH routinely in the future?* *g_admin2_q8; SAS:A2Q8*

Would you like to use HEMOPATCH routinely in the future?* *i_admin2_q8; text1; SAS:A2Q8*	∇ *Y* Yes ∇ *N* No *c_ny; text; SAS:$NY; display.style radio* *ARC arc_default*
If No, please specify reason:* *i_admin2_q8A; text100; SAS:A2Q8A* *ENABLE i_admin2_q8A if i_admin2_q8 = N*	_ *ARC arc_default*

Thank you for your participation. The information you provided will be used for statistical evaluation to assess the experiences gained from application of HEMOPATCH Sealing Haemostat.* *g_admin2_info; SAS:A2INFO*

Surgeon Initials* *i_admin2_inv; text5; SAS:A2INFO*	_ _ _ _ _ *ARC arc_default*
Completion Date* *i_admin2_date; date10; SAS:A2DATE*	_ _ /_ _ /_ _ _ _ mm/dd/yyyy *ARC arc_default*

HEMOPATCH Questionnaire Surgeon 2: {0} {1} *f_admin2_2surgeon; EP(0): ItemGroupData[@OID="g_admin2_surgid"]/ItemData[@OID="i_admin2_nam1"]; EP(1): ItemGroupData[@OID="g_admin2_surgid"]/ItemData[@OID="i_admin2_nam2"]*

HEMOPATCH Intraoperative Use Evaluation Questionnaire* *g_admin2_surgid; SAS:A2SRGID*

First Name:* *i_admin2_nam1; text20; SAS:A2NAM1*	_ *ARC arc_default*
Last Name:* *i_admin2_nam2; text20; SAS:A2NAM2*	_ *ARC arc_default*
Title:* *i_admin2_nam3; text20; SAS:A2NAM3*	_ *ARC arc_default*
Hospital and Department* *i_admin2_Hosp; text200; SAS:A2HOSP*	_ *ARC arc_default*

HEMOPATCH Intraoperative Use Evaluation Questionnaire* g_admin2_surgid; SAS:A2SRGID

How many times have you applied HEMOPATCH Sealing Haemostat before?* i_admin2_hemo; text9; SAS:A2HEMO	∇ 0 0 ∇ 1 1 ∇ 2 2 ∇ 3 3 ∇ 4 or more 4 or more c_app; text; SAS:$app<br>ARC arc_default
Have you been trained by Baxter on the product?* i_admin2_train; text1; SAS:A2TRAIN	∇ Y Yes ∇ N No c_ny; text; SAS:$NY; display.style radio<br>ARC arc_default

Could you please rate HEMOPATCH on the following features?* g_admin2_q5; SAS:A2Q5

Ease of preparation* i_admin2_q5_1; text50; SAS:A2Q5_1	∇ EXCELLENT Excellent ∇ GOOD Good ∇ MODERATE Moderate ∇ POOR Poor ∇ NOT APPLICABLE Not Applicable c_feat; text; SAS:$FEAT; display.style radio<br>ARC arc_default
Ease of handling* i_admin2_q5_2; text50; SAS:A2Q5_2	∇ EXCELLENT Excellent ∇ GOOD Good ∇ MODERATE Moderate ∇ POOR Poor ∇ NOT APPLICABLE Not Applicable c_feat; text; SAS:$FEAT; display.style radio<br>ARC arc_default
Tissue adherence* i_admin2_q5_3; text50; SAS:A2Q5_3	∇ EXCELLENT Excellent ∇ GOOD Good ∇ MODERATE Moderate ∇ POOR Poor ∇ NOT APPLICABLE Not Applicable c_feat; text; SAS:$FEAT; display.style radio<br>ARC arc_default
Flexibility / Pliability* i_admin2_q5_4; text50; SAS:A2Q5_4	∇ EXCELLENT Excellent ∇ GOOD Good ∇ MODERATE Moderate ∇ POOR Poor ∇ NOT APPLICABLE Not Applicable c_feat; text; SAS:$FEAT; display.style radio<br>ARC arc_default
Haemostatic efficacy* i_admin2_q5_5; text50; SAS:A2Q5_5	∇ EXCELLENT Excellent ∇ GOOD Good ∇ MODERATE Moderate ∇ POOR Poor ∇ NOT APPLICABLE Not Applicable c_feat; text; SAS:$FEAT; display.style radio<br>ARC arc_default
Surgeon overall satisfaction* i_admin2_q5_6; text50; SAS:A2Q5_6	∇ EXCELLENT Excellent ∇ GOOD Good ∇ MODERATE Moderate ∇ POOR Poor ∇ NOT APPLICABLE Not Applicable c_feat; text; SAS:$FEAT; display.style radio<br>ARC arc_default

What other haemostatic patches have you used before (check all that apply):* g_admin2_q6; SAS:A2Q6

Tachosil Patch i_admin2_q6_1; text1; SAS:A2Q6_1	☑ Y c_checkyes; text; SAS:$CHKYES; display.style checkbox<br>ARC arc_default
Collagen Patch (e.g. TissuFleece, GentaFleece) i_admin2_q6_2; text1; SAS:A2Q6_2	☑ Y c_checkyes; text; SAS:$CHKYES; display.style checkbox<br>ARC arc_default
Oxidized Cellulose Patch (e.g. Surgicel) i_admin2_q6_3; text1; SAS:A2Q6_3	☑ Y c_checkyes; text; SAS:$CHKYES; display.style checkbox<br>ARC arc_default
Flowable Matrix (e.g. Floseal, Surgiflo) i_admin2_q6_4; text1; SAS:A2Q6_4	☑ Y c_checkyes; text; SAS:$CHKYES; display.style checkbox<br>ARC arc_default
Sealant (e.g. Tisseel) i_admin2_q6_5; text1; SAS:A2Q6_5	☑ Y c_checkyes; text; SAS:$CHKYES; display.style checkbox<br>ARC arc_default
Other i_admin2_q6_6; text1; SAS:A2Q6_6	☑ Y c_checkyes; text; SAS:$CHKYES; display.style checkbox<br>ARC arc_default
Other, Specify* i_admin2_q6_6A; text50; SAS:A2Q6_6A<br>ENABLE i_admin2_q6_6A if i_admin2_q6_6 = Y	_ ARC arc_default
None i_admin2_q6_7; text1; SAS:A2Q6_7	☑ Y c_checkyes; text; SAS:$CHKYES; display.style checkbox<br>ARC arc_default

Compared to other haemostatic patches you have used, could you please rate HEMOPATCH on the following features? (Please tick only one box per line)* *g_admin2_q7; SAS:A2Q7*

Ease of preparation* *i_admin2_q7_1; text50; SAS:A2Q7_1*	∇ MUCH BETTER Much Better ∇ BETTER Better ∇ EQUIVALENT Equivalent ∇ WORSE Worse ∇ NOT APPLICABLE Not Applicable *c_feats; text; SAS:$FEATS; display.style radio* ARC arc_default
Ease of handling* *i_admin2_q7_2; text50; SAS:A2Q7_2*	∇ MUCH BETTER Much Better ∇ BETTER Better ∇ EQUIVALENT Equivalent ∇ WORSE Worse ∇ NOT APPLICABLE Not Applicable *c_feats; text; SAS:$FEATS; display.style radio* ARC arc_default
Flexibility / Pliability* *i_admin2_q7_3; text50; SAS:A2Q7_3*	∇ MUCH BETTER Much Better ∇ BETTER Better ∇ EQUIVALENT Equivalent ∇ WORSE Worse ∇ NOT APPLICABLE Not Applicable *c_feats; text; SAS:$FEATS; display.style radio* ARC arc_default
Tissue adherence* *i_admin2_q7_4; text50; SAS:A2Q7_4*	∇ MUCH BETTER Much Better ∇ BETTER Better ∇ EQUIVALENT Equivalent ∇ WORSE Worse ∇ NOT APPLICABLE Not Applicable *c_feats; text; SAS:$FEATS; display.style radio* ARC arc_default
Haemostatic efficacy* *i_admin2_q7_5; text50; SAS:A2Q7_5*	∇ MUCH BETTER Much Better ∇ BETTER Better ∇ EQUIVALENT Equivalent ∇ WORSE Worse ∇ NOT APPLICABLE Not Applicable *c_feats; text; SAS:$FEATS; display.style radio* ARC arc_default
Surgeon overall satisfaction* *i_admin2_q7_6; text50; SAS:A2Q7_6*	∇ MUCH BETTER Much Better ∇ BETTER Better ∇ EQUIVALENT Equivalent ∇ WORSE Worse ∇ NOT APPLICABLE Not Applicable *c_feats; text; SAS:$FEATS; display.style radio* ARC arc_default

Would you like to use HEMOPATCH routinely in the future?* *g_admin2_q8; SAS:A2Q8*

Would you like to use HEMOPATCH routinely in the future?* *i_admin2_q8; text1; SAS:A2Q8*	∇ Y Yes ∇ N No *c_ny; text; SAS:$NY; display.style radio* ARC arc_default
If No, please specify reason:* *i_admin2_q8A; text100; SAS:A2Q8A* ENABLE i_admin2_q8A if i_admin2_q8 = N	_ _<br>_ _<br>_ _<br>_ _<br>_ _ _ _ _ _ _ _ ARC arc_default

Thank you for your participation. The information you provided will be used for statistical evaluation to assess the experiences gained from application of HEMOPATCH Sealing Haemostat.* *g_admin2_info; SAS:A2INFO*

Surgeon Initials* *i_admin2_inv; text5; SAS:A2INFO*	_ _ _ _ _ ARC arc_default
Completion Date* *i_admin2_date; date10; SAS:A2DATE*	_ _ /_ _ /_ _ _ _ mm/dd/yyyy ARC arc_default

HEMOPATCH Questionnaire Surgeon 3: {0} {1} *f_admin2_3surgeon; EP(0): ItemGroupData[@OID="g_admin2_surgid"]/ItemData[@OID="i_admin2_nam1"]; EP(1): ItemGroupData[@OID="g_admin2_surgid"]/ItemData[@OID="i_admin2_nam2"]*

HEMOPATCH Intraoperative Use Evaluation Questionnaire* *g_admin2_surgid; SAS:A2SRGID*

First Name:* *i_admin2_nam1; text20; SAS:A2NAM1*	_ ARC arc_default

HEMOPATCH Intraoperative Use Evaluation Questionnaire* g_admin2_surgid; SAS:A2SRGID

Last Name:* i_admin2_nam2; text20; SAS:A2NAM2	_ ARC arc_default
Title:* i_admin2_nam3; text20; SAS:A2NAM3	_ ARC arc_default
Hospital and Department* i_admin2_Hosp; text200; SAS:A2HOSP	_ ARC arc_default
How many times have you applied HEMOPATCH Sealing Haemostat before?* i_admin2_hemo; text9; SAS:A2HEMO	∇ 0 0 ∇ 1 1 ∇ 2 2 ∇ 3 3 ∇ 4 or more 4 or more c_app; text; SAS:$app ARC arc_default
Have you been trained by Baxter on the product?* i_admin2_train; text1; SAS:A2TRAIN	∇ Y Yes ∇ N No c_ny; text; SAS:$NY; display.style radio ARC arc_default

Could you please rate HEMOPATCH on the following features?* g_admin2_q5; SAS:A2Q5

Ease of preparation* i_admin2_q5_1; text50; SAS:A2Q5_1	∇ EXCELLENT Excellent ∇ GOOD Good ∇ MODERATE Moderate ∇ POOR Poor ∇ NOT APPLICABLE Not Applicable c_feat; text; SAS:$FEAT; display.style radio ARC arc_default
Ease of handling* i_admin2_q5_2; text50; SAS:A2Q5_2	∇ EXCELLENT Excellent ∇ GOOD Good ∇ MODERATE Moderate ∇ POOR Poor ∇ NOT APPLICABLE Not Applicable c_feat; text; SAS:$FEAT; display.style radio ARC arc_default
Tissue adherence* i_admin2_q5_3; text50; SAS:A2Q5_3	∇ EXCELLENT Excellent ∇ GOOD Good ∇ MODERATE Moderate ∇ POOR Poor ∇ NOT APPLICABLE Not Applicable c_feat; text; SAS:$FEAT; display.style radio ARC arc_default
Flexibility / Pliability* i_admin2_q5_4; text50; SAS:A2Q5_4	∇ EXCELLENT Excellent ∇ GOOD Good ∇ MODERATE Moderate ∇ POOR Poor ∇ NOT APPLICABLE Not Applicable c_feat; text; SAS:$FEAT; display.style radio ARC arc_default
Haemostatic efficacy* i_admin2_q5_5; text50; SAS:A2Q5_5	∇ EXCELLENT Excellent ∇ GOOD Good ∇ MODERATE Moderate ∇ POOR Poor ∇ NOT APPLICABLE Not Applicable c_feat; text; SAS:$FEAT; display.style radio ARC arc_default
Surgeon overall satisfaction* i_admin2_q5_6; text50; SAS:A2Q5_6	∇ EXCELLENT Excellent ∇ GOOD Good ∇ MODERATE Moderate ∇ POOR Poor ∇ NOT APPLICABLE Not Applicable c_feat; text; SAS:$FEAT; display.style radio ARC arc_default

What other haemostatic patches have you used before (check all that apply):* g_admin2_q6; SAS:A2Q6

Tachosil Patch i_admin2_q6_1; text1; SAS:A2Q6_1	☑ Y c_checkyes; text; SAS:$CHKYES; display.style checkbox ARC arc_default
Collagen Patch (e.g. TissuFleece, GentaFleece) i_admin2_q6_2; text1; SAS:A2Q6_2	☑ Y c_checkyes; text; SAS:$CHKYES; display.style checkbox ARC arc_default
Oxidized Cellulose Patch (e.g. Surgicel) i_admin2_q6_3; text1; SAS:A2Q6_3	☑ Y c_checkyes; text; SAS:$CHKYES; display.style checkbox ARC arc_default

What other haemostatic patches have you used before (check all that apply):* *g_admin2_q6; SAS:A2Q6*

Flowable Matrix (e.g. Floseal, Surgiflo) *i_admin2_q6_4; text1; SAS:A2Q6_4*	☑ *Y c_checkyes; text; SAS:$CHKYES; display.style checkbox* ARC arc_default
Sealant (e.g. Tisseel) *i_admin2_q6_5; text1; SAS:A2Q6_5*	☑ *Y c_checkyes; text; SAS:$CHKYES; display.style checkbox* ARC arc_default
Other *i_admin2_q6_6; text1; SAS:A2Q6_6*	☑ *Y c_checkyes; text; SAS:$CHKYES; display.style checkbox* ARC arc_default
Other, Specify* *i_admin2_q6_6A; text50; SAS:A2Q6_6A* ENABLE i_admin2_q6_6A if i_admin2_q6_6 = Y	________________________ ________________________ _____ ARC arc_default
None *i_admin2_q6_7; text1; SAS:A2Q6_7*	☑ *Y c_checkyes; text; SAS:$CHKYES; display.style checkbox* ARC arc_default

Compared to other haemostatic patches you have used, could you please rate HEMOPATCH on the following features? (Please tick only one box per line)* *g_admin2_q7; SAS:A2Q7*

Ease of preparation* *i_admin2_q7_1; text50; SAS:A2Q7_1*	∇ MUCH BETTER Much Better ∇ BETTER Better ∇ EQUIVALENT Equivalent ∇ WORSE Worse ∇ NOT APPLICABLE Not Applicable *c_feats; text; SAS:$FEATS; display.style radio* ARC arc_default
Ease of handling* *i_admin2_q7_2; text50; SAS:A2Q7_2*	∇ MUCH BETTER Much Better ∇ BETTER Better ∇ EQUIVALENT Equivalent ∇ WORSE Worse ∇ NOT APPLICABLE Not Applicable *c_feats; text; SAS:$FEATS; display.style radio* ARC arc_default
Flexibility / Pliability* *i_admin2_q7_3; text50; SAS:A2Q7_3*	∇ MUCH BETTER Much Better ∇ BETTER Better ∇ EQUIVALENT Equivalent ∇ WORSE Worse ∇ NOT APPLICABLE Not Applicable *c_feats; text; SAS:$FEATS; display.style radio* ARC arc_default
Tissue adherence* *i_admin2_q7_4; text50; SAS:A2Q7_4*	∇ MUCH BETTER Much Better ∇ BETTER Better ∇ EQUIVALENT Equivalent ∇ WORSE Worse ∇ NOT APPLICABLE Not Applicable *c_feats; text; SAS:$FEATS; display.style radio* ARC arc_default
Haemostatic efficacy* *i_admin2_q7_5; text50; SAS:A2Q7_5*	∇ MUCH BETTER Much Better ∇ BETTER Better ∇ EQUIVALENT Equivalent ∇ WORSE Worse ∇ NOT APPLICABLE Not Applicable *c_feats; text; SAS:$FEATS; display.style radio* ARC arc_default
Surgeon overall satisfaction* *i_admin2_q7_6; text50; SAS:A2Q7_6*	∇ MUCH BETTER Much Better ∇ BETTER Better ∇ EQUIVALENT Equivalent ∇ WORSE Worse ∇ NOT APPLICABLE Not Applicable *c_feats; text; SAS:$FEATS; display.style radio* ARC arc_default

Would you like to use HEMOPATCH routinely in the future?* *g_admin2_q8; SAS:A2Q8*

Would you like to use HEMOPATCH routinely in the future?* *i_admin2_q8; text1; SAS:A2Q8*	∇ Y Yes ∇ N No *c_ny; text; SAS:$NY; display.style radio* ARC arc_default
If No, please specify reason:* *i_admin2_q8A; text100; SAS:A2Q8A* ENABLE i_admin2_q8A if i_admin2_q8 = N	________________________ ________________________ ________________________ ________________________ ________ ARC arc_default

Thank you for your participation. The information you provided will be used for statistical evaluation to assess the experiences gained from application of HEMOPATCH Sealing Haemostat.* *g_admin2_info; SAS:A2INFO*

Surgeon Initials* *i_admin2_inv; text5; SAS:A2INFO*	_ _ _ _ _ *ARC arc_default*
Completion Date* *i_admin2_date; date10; SAS:A2DATE*	_ _ /_ _ /_ _ _ _ mm/dd/yyyy *ARC arc_default*

HEMOPATCH Questionnaire Surgeon 4: {0} {1} *f_admin2_4surgeon; EP(0): ItemGroupData[@OID="g_admin2_surgid"]/ItemData[@OID="i_admin2_nam1"]; EP(1): ItemGroupData[@OID="g_admin2_surgid"]/ItemData[@OID="i_admin2_nam2"]*

HEMOPATCH Intraoperative Use Evaluation Questionnaire* *g_admin2_surgid; SAS:A2SRGID*

First Name:* *i_admin2_nam1; text20; SAS:A2NAM1*	_ *ARC arc_default*
Last Name:* *i_admin2_nam2; text20; SAS:A2NAM2*	_ *ARC arc_default*
Title:* *i_admin2_nam3; text20; SAS:A2NAM3*	_ *ARC arc_default*
Hospital and Department* *i_admin2_Hosp; text200; SAS:A2HOSP*	_ (multiple lines) *ARC arc_default*
How many times have you applied HEMOPATCH Sealing Haemostat before?* *i_admin2_hemo; text9; SAS:A2HEMO*	∇ 0 0 ∇ 1 1 ∇ 2 2 ∇ 3 3 ∇ 4 or more 4 or more *c_app; text; SAS:$app* *ARC arc_default*
Have you been trained by Baxter on the product?* *i_admin2_train; text1; SAS:A2TRAIN*	∇ Y Yes ∇ N No *c_ny; text; SAS:$NY; display.style radio* *ARC arc_default*

Could you please rate HEMOPATCH on the following features?* *g_admin2_q5; SAS:A2Q5*

Ease of preparation* *i_admin2_q5_1; text50; SAS:A2Q5_1*	∇ EXCELLENT Excellent ∇ GOOD Good ∇ MODERATE Moderate ∇ POOR Poor ∇ NOT APPLICABLE Not Applicable *c_feat; text; SAS:$FEAT; display.style radio* *ARC arc_default*
Ease of handling* *i_admin2_q5_2; text50; SAS:A2Q5_2*	∇ EXCELLENT Excellent ∇ GOOD Good ∇ MODERATE Moderate ∇ POOR Poor ∇ NOT APPLICABLE Not Applicable *c_feat; text; SAS:$FEAT; display.style radio* *ARC arc_default*
Tissue adherence* *i_admin2_q5_3; text50; SAS:A2Q5_3*	∇ EXCELLENT Excellent ∇ GOOD Good ∇ MODERATE Moderate ∇ POOR Poor ∇ NOT APPLICABLE Not Applicable *c_feat; text; SAS:$FEAT; display.style radio* *ARC arc_default*
Flexibility / Pliability* *i_admin2_q5_4; text50; SAS:A2Q5_4*	∇ EXCELLENT Excellent ∇ GOOD Good ∇ MODERATE Moderate ∇ POOR Poor ∇ NOT APPLICABLE Not Applicable *c_feat; text; SAS:$FEAT; display.style radio* *ARC arc_default*

Could you please rate HEMOPATCH on the following features?* *g_admin2_q5; SAS:A2Q5*

Haemostatic efficacy* *i_admin2_q5_5; text50; SAS:A2Q5_5*	∇ EXCELLENT Excellent ∇ GOOD Good ∇ MODERATE Moderate ∇ POOR Poor ∇ NOT APPLICABLE Not Applicable *c_feat; text; SAS:$FEAT; display.style radio* ARC arc_default
Surgeon overall satisfaction* *i_admin2_q5_6; text50; SAS:A2Q5_6*	∇ EXCELLENT Excellent ∇ GOOD Good ∇ MODERATE Moderate ∇ POOR Poor ∇ NOT APPLICABLE Not Applicable *c_feat; text; SAS:$FEAT; display.style radio* ARC arc_default

What other haemostatic patches have you used before (check all that apply):* *g_admin2_q6; SAS:A2Q6*

Tachosil Patch *i_admin2_q6_1; text1; SAS:A2Q6_1*	☑ Y *c_checkyes; text; SAS:$CHKYES; display.style checkbox* ARC arc_default
Collagen Patch (e.g. TissuFleece, GentaFleece) *i_admin2_q6_2; text1; SAS:A2Q6_2*	☑ Y *c_checkyes; text; SAS:$CHKYES; display.style checkbox* ARC arc_default
Oxidized Cellulose Patch (e.g. Surgicel) *i_admin2_q6_3; text1; SAS:A2Q6_3*	☑ Y *c_checkyes; text; SAS:$CHKYES; display.style checkbox* ARC arc_default
Flowable Matrix (e.g. Floseal, Surgiflo) *i_admin2_q6_4; text1; SAS:A2Q6_4*	☑ Y *c_checkyes; text; SAS:$CHKYES; display.style checkbox* ARC arc_default
Sealant (e.g. Tisseel) *i_admin2_q6_5; text1; SAS:A2Q6_5*	☑ Y *c_checkyes; text; SAS:$CHKYES; display.style checkbox* ARC arc_default
Other *i_admin2_q6_6; text1; SAS:A2Q6_6*	☑ Y *c_checkyes; text; SAS:$CHKYES; display.style checkbox* ARC arc_default
Other, Specify* *i_admin2_q6_6A; text50; SAS:A2Q6_6A* ENABLE i_admin2_q6_6A if i_admin2_q6_6 = Y	______________________________ ARC arc_default
None *i_admin2_q6_7; text1; SAS:A2Q6_7*	☑ Y *c_checkyes; text; SAS:$CHKYES; display.style checkbox* ARC arc_default

Compared to other haemostatic patches you have used, could you please rate HEMOPATCH on the following features? (Please tick only one box per line)* *g_admin2_q7; SAS:A2Q7*

Ease of preparation* *i_admin2_q7_1; text50; SAS:A2Q7_1*	∇ MUCH BETTER Much Better ∇ BETTER Better ∇ EQUIVALENT Equivalent ∇ WORSE Worse ∇ NOT APPLICABLE Not Applicable *c_feats; text; SAS:$FEATS; display.style radio* ARC arc_default
Ease of handling* *i_admin2_q7_2; text50; SAS:A2Q7_2*	∇ MUCH BETTER Much Better ∇ BETTER Better ∇ EQUIVALENT Equivalent ∇ WORSE Worse ∇ NOT APPLICABLE Not Applicable *c_feats; text; SAS:$FEATS; display.style radio* ARC arc_default
Flexibility / Pliability* *i_admin2_q7_3; text50; SAS:A2Q7_3*	∇ MUCH BETTER Much Better ∇ BETTER Better ∇ EQUIVALENT Equivalent ∇ WORSE Worse ∇ NOT APPLICABLE Not Applicable *c_feats; text; SAS:$FEATS; display.style radio* ARC arc_default
Tissue adherence* *i_admin2_q7_4; text50; SAS:A2Q7_4*	∇ MUCH BETTER Much Better ∇ BETTER Better ∇ EQUIVALENT Equivalent ∇ WORSE Worse ∇ NOT APPLICABLE Not Applicable *c_feats; text; SAS:$FEATS; display.style radio* ARC arc_default
Haemostatic efficacy* *i_admin2_q7_5; text50; SAS:A2Q7_5*	∇ MUCH BETTER Much Better ∇ BETTER Better ∇ EQUIVALENT Equivalent ∇ WORSE Worse ∇ NOT APPLICABLE Not Applicable *c_feats; text; SAS:$FEATS; display.style radio* ARC arc_default

Compared to other haemostatic patches you have used, could you please rate HEMOPATCH on the following features? (Please tick only one box per line)* *g_admin2_q7; SAS:A2Q7*

Surgeon overall satisfaction* *i_admin2_q7_6; text50; SAS:A2Q7_6*	∇ *MUCH BETTER* Much Better ∇ *BETTER* Better ∇ *EQUIVALENT* Equivalent ∇ *WORSE* Worse ∇ *NOT APPLICABLE* Not Applicable *c_feats; text; SAS:$FEATS; display.style radio* ARC arc_default

Would you like to use HEMOPATCH routinely in the future?* *g_admin2_q8; SAS:A2Q8*

Would you like to use HEMOPATCH routinely in the future?* *i_admin2_q8; text1; SAS:A2Q8*	∇ *Y* Yes ∇ *N* No *c_ny; text; SAS:$NY; display.style radio* ARC arc_default
If No, please specify reason:* *i_admin2_q8A; text100; SAS:A2Q8A* ENABLE i_admin2_q8A if i_admin2_q8 = N	_ _<br>_ _<br>_ _<br>_ _<br>_ _ _ _ _ _ _ _ ARC arc_default

Thank you for your participation. The information you provided will be used for statistical evaluation to assess the experiences gained from application of HEMOPATCH Sealing Haemostat.* *g_admin2_info; SAS:A2INFO*

Surgeon Initials* *i_admin2_inv; text5; SAS:A2INFO*	_ _ _ _ _ ARC arc_default
Completion Date* *i_admin2_date; date10; SAS:A2DATE*	_ _ /_ _ /_ _ _ _ mm/dd/yyyy ARC arc_default

HEMOPATCH Questionnaire Surgeon 5: {0} {1} *f_admin2_5surgeon; EP(0): ItemGroupData[@OID="g_admin2_surgid"]/ItemData[@OID="i_admin2_nam1"]; EP(1): ItemGroupData[@OID="g_admin2_surgid"]/ItemData[@OID="i_admin2_nam2"]*

HEMOPATCH Intraoperative Use Evaluation Questionnaire* *g_admin2_surgid; SAS:A2SRGID*

First Name:* *i_admin2_nam1; text20; SAS:A2NAM1*	_ ARC arc_default
Last Name:* *i_admin2_nam2; text20; SAS:A2NAM2*	_ ARC arc_default
Title:* *i_admin2_nam3; text20; SAS:A2NAM3*	_ ARC arc_default
Hospital and Department* *i_admin2_Hosp; text200; SAS:A2HOSP*	_ _<br>_ _<br>_ _<br>_ _<br>_ _<br>_ _<br>_ _<br>_ _<br>_ _ _ _ _ _ _ _ _ _ _ _ _ _ _ _ ARC arc_default
How many times have you applied HEMOPATCH Sealing Haemostat before?* *i_admin2_hemo; text9; SAS:A2HEMO*	∇ *0* 0 ∇ *1* 1 ∇ *2* 2 ∇ *3* 3 ∇ *4 or more* 4 or more *c_app; text; SAS:$app* ARC arc_default
Have you been trained by Baxter on the product?* *i_admin2_train; text1; SAS:A2TRAIN*	∇ *Y* Yes ∇ *N* No *c_ny; text; SAS:$NY; display.style radio* ARC arc_default

Could you please rate HEMOPATCH on the following features?* *g_admin2_q5; SAS:A2Q5*

Could you please rate HEMOPATCH on the following features?* *g_admin2_q5; SAS:A2Q5*

Ease of preparation* *i_admin2_q5_1; text50; SAS:A2Q5_1*	∇ EXCELLENT Excellent ∇ GOOD Good ∇ MODERATE Moderate ∇ POOR Poor ∇ NOT APPLICABLE Not Applicable *c_feat; text; SAS:$FEAT; display.style radio* *ARC arc_default*
Ease of handling* *i_admin2_q5_2; text50; SAS:A2Q5_2*	∇ EXCELLENT Excellent ∇ GOOD Good ∇ MODERATE Moderate ∇ POOR Poor ∇ NOT APPLICABLE Not Applicable *c_feat; text; SAS:$FEAT; display.style radio* *ARC arc_default*
Tissue adherence* *i_admin2_q5_3; text50; SAS:A2Q5_3*	∇ EXCELLENT Excellent ∇ GOOD Good ∇ MODERATE Moderate ∇ POOR Poor ∇ NOT APPLICABLE Not Applicable *c_feat; text; SAS:$FEAT; display.style radio* *ARC arc_default*
Flexibility / Pliability* *i_admin2_q5_4; text50; SAS:A2Q5_4*	∇ EXCELLENT Excellent ∇ GOOD Good ∇ MODERATE Moderate ∇ POOR Poor ∇ NOT APPLICABLE Not Applicable *c_feat; text; SAS:$FEAT; display.style radio* *ARC arc_default*
Haemostatic efficacy* *i_admin2_q5_5; text50; SAS:A2Q5_5*	∇ EXCELLENT Excellent ∇ GOOD Good ∇ MODERATE Moderate ∇ POOR Poor ∇ NOT APPLICABLE Not Applicable *c_feat; text; SAS:$FEAT; display.style radio* *ARC arc_default*
Surgeon overall satisfaction* *i_admin2_q5_6; text50; SAS:A2Q5_6*	∇ EXCELLENT Excellent ∇ GOOD Good ∇ MODERATE Moderate ∇ POOR Poor ∇ NOT APPLICABLE Not Applicable *c_feat; text; SAS:$FEAT; display.style radio* *ARC arc_default*

What other haemostatic patches have you used before (check all that apply):* *g_admin2_q6; SAS:A2Q6*

Tachosil Patch *i_admin2_q6_1; text1; SAS:A2Q6_1*	☑ Y *c_checkyes; text; SAS:$CHKYES; display.style checkbox* *ARC arc_default*
Collagen Patch (e.g. TissuFleece, GentaFleece) *i_admin2_q6_2; text1; SAS:A2Q6_2*	☑ Y *c_checkyes; text; SAS:$CHKYES; display.style checkbox* *ARC arc_default*
Oxidized Cellulose Patch (e.g. Surgicel) *i_admin2_q6_3; text1; SAS:A2Q6_3*	☑ Y *c_checkyes; text; SAS:$CHKYES; display.style checkbox* *ARC arc_default*
Flowable Matrix (e.g. Floseal, Surgiflo) *i_admin2_q6_4; text1; SAS:A2Q6_4*	☑ Y *c_checkyes; text; SAS:$CHKYES; display.style checkbox* *ARC arc_default*
Sealant (e.g. Tisseel) *i_admin2_q6_5; text1; SAS:A2Q6_5*	☑ Y *c_checkyes; text; SAS:$CHKYES; display.style checkbox* *ARC arc_default*
Other *i_admin2_q6_6; text1; SAS:A2Q6_6*	☑ Y *c_checkyes; text; SAS:$CHKYES; display.style checkbox* *ARC arc_default*
Other, Specify* *i_admin2_q6_6A; text50; SAS:A2Q6_6A* *ENABLE i_admin2_q6_6A if i_admin2_q6_6 = Y*	_ *ARC arc_default*
None *i_admin2_q6_7; text1; SAS:A2Q6_7*	☑ Y *c_checkyes; text; SAS:$CHKYES; display.style checkbox* *ARC arc_default*

Compared to other haemostatic patches you have used, could you please rate HEMOPATCH on the following features? (Please tick only one box per line)* *g_admin2_q7; SAS:A2Q7*

Ease of preparation* *i_admin2_q7_1; text50; SAS:A2Q7_1*	∇ MUCH BETTER Much Better ∇ BETTER Better ∇ EQUIVALENT Equivalent ∇ WORSE Worse ∇ NOT APPLICABLE Not Applicable *c_feats; text; SAS:$FEATS; display.style radio* *ARC arc_default*

Compared to other haemostatic patches you have used, could you please rate HEMOPATCH on the following features? (Please tick only one box per line)* *g_admin2_q7; SAS:A2Q7*

Ease of handling* *i_admin2_q7_2; text50; SAS:A2Q7_2*	∇ *MUCH BETTER* Much Better ∇ *BETTER* Better ∇ *EQUIVALENT* Equivalent ∇ *WORSE* Worse ∇ *NOT APPLICABLE* Not Applicable *c_feats; text; SAS:$FEATS; display.style radio* *ARC arc_default*
Flexibility / Pliability* *i_admin2_q7_3; text50; SAS:A2Q7_3*	∇ *MUCH BETTER* Much Better ∇ *BETTER* Better ∇ *EQUIVALENT* Equivalent ∇ *WORSE* Worse ∇ *NOT APPLICABLE* Not Applicable *c_feats; text; SAS:$FEATS; display.style radio* *ARC arc_default*
Tissue adherence* *i_admin2_q7_4; text50; SAS:A2Q7_4*	∇ *MUCH BETTER* Much Better ∇ *BETTER* Better ∇ *EQUIVALENT* Equivalent ∇ *WORSE* Worse ∇ *NOT APPLICABLE* Not Applicable *c_feats; text; SAS:$FEATS; display.style radio* *ARC arc_default*
Haemostatic efficacy* *i_admin2_q7_5; text50; SAS:A2Q7_5*	∇ *MUCH BETTER* Much Better ∇ *BETTER* Better ∇ *EQUIVALENT* Equivalent ∇ *WORSE* Worse ∇ *NOT APPLICABLE* Not Applicable *c_feats; text; SAS:$FEATS; display.style radio* *ARC arc_default*
Surgeon overall satisfaction* *i_admin2_q7_6; text50; SAS:A2Q7_6*	∇ *MUCH BETTER* Much Better ∇ *BETTER* Better ∇ *EQUIVALENT* Equivalent ∇ *WORSE* Worse ∇ *NOT APPLICABLE* Not Applicable *c_feats; text; SAS:$FEATS; display.style radio* *ARC arc_default*

Would you like to use HEMOPATCH routinely in the future?* *g_admin2_q8; SAS:A2Q8*

Would you like to use HEMOPATCH routinely in the future?* *i_admin2_q8; text1; SAS:A2Q8*	∇ *Y* Yes ∇ *N* No *c_ny; text; SAS:$NY; display.style radio* *ARC arc_default*
If No, please specify reason:* *i_admin2_q8A; text100; SAS:A2Q8A* *ENABLE i_admin2_q8A if i_admin2_q8 = N*	_ *ARC arc_default*

Thank you for your participation. The information you provided will be used for statistical evaluation to assess the experiences gained from application of HEMOPATCH Sealing Haemostat.* *g_admin2_info; SAS:A2INFO*

Surgeon Initials* *i_admin2_inv; text5; SAS:A2INFO*	_ _ _ _ _ *ARC arc_default*
Completion Date* *i_admin2_date; date10; SAS:A2DATE*	_ _ /_ _ /_ _ _ _ mm/dd/yyyy *ARC arc_default*

New, redefined or unused Definitions

New, redefined or unused Forms

HEMOPATCH Questionnaire Surgeon 1: {1} {2} [# _ _] *f_admin2_surgeon; EP(0): ItemGroupData[@OID="g_admin2_surgid"]/ItemData[@OID="i_admin2_nam1"]; EP(1): ItemGroupData[@OID="g_admin2_surgid"]/ItemData[@OID="i_admin2_nam2"]*

New, redefined or unused ItemGroups

If "Yes", please click 'Add ItemGroup' to record each product complaint below: [# _ _] *g_pc; SAS:PC; display.style horizontal*

Registration *g_register; SAS:REGISTER*

New, redefined or unused Items

HEMOPATCH Questionnaire #: *i_admin2_survid; integer1; SAS:A2SURVID*

Is the event an Adverse Device Event (ADE)? *i_aeade; text1; SAS:AEADE*

Calculated Age - Years (Derived, hidden) *i_agecalc; integer3; SAS:AGECALC*

For newborns and infants, please specify the subject's Age at the time of Informed Consent. ***TO BE HIDDEN/DELETED *i_ageinf; text3; SAS:AGEINF*

Age (months) *i_agemo; integer2; SAS:AGEMO*

Date of Birth - Day ***TO BE HIDDEN/DELETED *i_brthdy; text2; SAS:BRTHDY*

Date of Birth - Month ***TO BE HIDDEN/DELETED *i_brthmo; text3; SAS:BRTHMO*

Date of Birth - Year ***TO BE HIDDEN/DELETED *i_brthyr; integer4; SAS:BRTHYR*

Stop Date (Derived) *i_cmendt; text11; SAS:CMENDT*

Start Date (Derived) *i_cmstdt; text11; SAS:CMSTDT*

End Date (Derived) *i_mhendat; text11; SAS:MHENDAT*

Start Date (Derived) *i_mhstdat; text11; SAS:MHSTDAT*

Was the information sent to the Product Complaint Department? *i_pcrepyn; text1; SAS:PCREPYN*

Protocol Version Date *i_protvdat; date10; SAS:PROTVDAT*

Registration Date *i_regdat; date10; SAS:REGDAT*

Registration ID (Derived, Hidden) *i_regid; text12; SAS:REGID*

Time of Informed Assent Signature: ***TO BE HIDDEN/DELETED *i_rfiatim; time5; SAS:RFIATIM*

Time of Informed Consent Signature: ***TO BE HIDDEN/DELETED *i_rfictim; time5; SAS:RFICTIM*

New, redefined or unused Codelists

c_aecat

c_aesi

c_aetyp

c_ageinf

c_calmon

c_patchreas

c_patchsp

c_prtyp

c_rficvers

New, redefined or unused Measurement Units

m_kg

m_lb

mu_hours

mu_min

mu_months

mu_weeks

mu_years

New, redefined or unused Actions

a_c_age

a_e_dm_005

a_q_CM009

a_q_DM001

a_q_DM004

a_q_DSIC001

a_q_DSIC002

a_q_DSIC003

a_q_MH005

a_q_SURGEON002

a_q_SURGERY003
